# A novel rare *CUBN* variant and three additional genes identified in Europeans with and without diabetes: results from an exome-wide association study of albuminuria

**DOI:** 10.1007/s00125-018-4783-z

**Published:** 2018-12-13

**Authors:** Tarunveer S. Ahluwalia, Christina-Alexandra Schulz, Johannes Waage, Tea Skaaby, Niina Sandholm, Natalie van Zuydam, Romain Charmet, Jette Bork-Jensen, Peter Almgren, Betina H. Thuesen, Mathilda Bedin, Ivan Brandslund, Cramer K. Christensen, Allan Linneberg, Emma Ahlqvist, Per-Henrik Groop, Samy Hadjadj, David-Alexandre Tregouet, Marit E. Jørgensen, Niels Grarup, Oluf Pedersen, Matias Simons, Leif Groop, Marju Orho-Melander, Mark I. McCarthy, Olle Melander, Peter Rossing, Tuomas O. Kilpeläinen, Torben Hansen

**Affiliations:** 1 Steno Diabetes Center Copenhagen, Niels Steensens Vej 2, 2820 Gentofte, Denmark; 2 Novo Nordisk Foundation Center for Basic Metabolic Research, Faculty of Health and Medical Sciences, University of Copenhagen, Copenhagen, Denmark; 3 Copenhagen Prospective Studies on Asthma in Childhood, Gentofte and Herlev Hospital, University of Copenhagen, Copenhagen, Denmark; 4 Department of Clinical Sciences Malmö, Lund University, Malmö, Sweden; 5 Center for Clinical Research and Prevention, Bispebjerg and Frederiksberg Hospital, Capital Region, Copenhagen, Denmark; 6 Folkhälsan Institute of Genetics, Folkhälsan Research Center, Helsinki, Finland; 7 Abdominal Center, Nephrology, University of Helsinki and Helsinki University Hospital, Helsinki, Finland; 8 Research Programs Unit, Diabetes and Obesity, University of Helsinki, Helsinki, Finland; 9 Wellcome Centre for Human Genetics, Nuffield Department of Medicine, University of Oxford, Oxford, UK; 10 Oxford Centre for Diabetes, Endocrinology and Metabolism, Radcliffe Department of Medicine, University of Oxford, Oxford, UK; 11 Inserm UMR-S 1166, Sorbonne Universités, UPMC Université Paris, Paris, France; 12 Paris Descartes University–Sorbonne Paris Cité, Imagine Institute, Paris, France; 13 Department of Clinical Immunology and Biochemistry, Lillebaelt Hospital, Vejle, Denmark; 14 Department of Internal Medicine and Endocrinology, Lillebaelt Hospital, Vejle, Denmark; 15 Department of Diabetes, Central Clinical School, Monash University, Melbourne, VIC Australia; 16 L’institut du thorax, Department of Endocrinology, CIC 1413 INSERM, CHU Nantes, Nantes, France; 17 National Institute of Public Health, University of Southern Denmark, Copenhagen, Denmark; 18 Institute for Molecular Medicine Finland (FIMM), University of Helsinki, Helsinki, Finland; 19 University of Copenhagen, Copenhagen, Denmark; 20 Faculty of Health Sciences, University of Southern Denmark, Odense, Denmark

**Keywords:** Albuminuria, Diabetes, DKD, Exome chip, Genetics, Genome-wide association study, Kidney disease, GWAS, Rare variant, SKAT, Type 2 diabetes

## Abstract

**Aims/hypothesis:**

Identifying rare coding variants associated with albuminuria may open new avenues for preventing chronic kidney disease and end-stage renal disease, which are highly prevalent in individuals with diabetes. Efforts to identify genetic susceptibility variants for albuminuria have so far been limited, with the majority of studies focusing on common variants.

**Methods:**

We performed an exome-wide association study to identify coding variants in a two-stage (discovery and replication) approach. Data from 33,985 individuals of European ancestry (15,872 with and 18,113 without diabetes) and 2605 Greenlanders were included.

**Results:**

We identified a rare (minor allele frequency [MAF]: 0.8%) missense (A1690V) variant in *CUBN* (rs141640975, β = 0.27, *p* = 1.3 × 10^−11^) associated with albuminuria as a continuous measure in the combined European meta-analysis. The presence of each rare allele of the variant was associated with a 6.4% increase in albuminuria. The rare *CUBN* variant had an effect that was three times stronger in individuals with type 2 diabetes compared with those without (*p*_interaction_ = 7.0 × 10^−4^, β with diabetes = 0.69, β without diabetes = 0.20) in the discovery meta-analysis. Gene-aggregate tests based on rare and common variants identified three additional genes associated with albuminuria (*HES1*, *CDC73* and *GRM5*) after multiple testing correction (*p*_Bonferroni_ < 2.7 × 10^−6^).

**Conclusions/interpretation:**

The current study identifies a rare coding variant in the *CUBN* locus and other potential genes associated with albuminuria in individuals with and without diabetes. These genes have been implicated in renal and cardiovascular dysfunction. The findings provide new insights into the genetic architecture of albuminuria and highlight target genes and pathways for the prevention of diabetes-related kidney disease.

**Electronic supplementary material:**

The online version of this article (10.1007/s00125-018-4783-z) contains peer-reviewed but unedited supplementary material, which is available to authorised users.

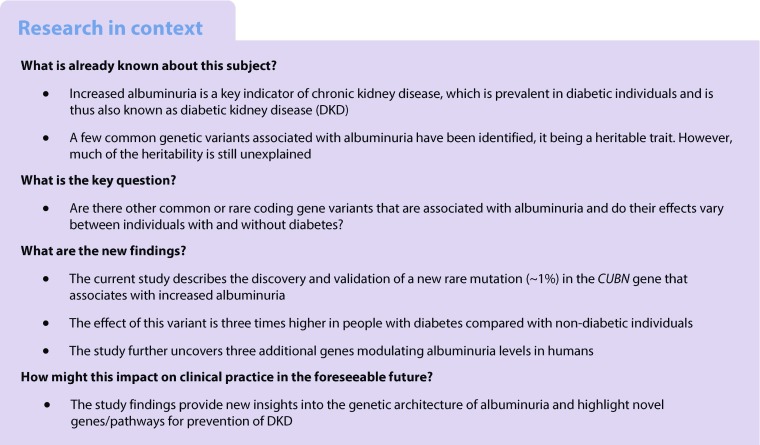



## Introduction

Albuminuria is a manifestation of chronic kidney disease (CKD), a major health burden worldwide with a current prevalence of 14.8% in the USA [1]. In individuals with CKD, changes in albuminuria are strongly associated with the risk of end-stage renal disease and death [2]. Diabetic individuals have an increased risk of developing CKD (referred to as diabetic kidney disease [DKD]); in the USA, the prevalence of CKD is ~41% among individuals with diabetes in comparison with ~10% in individuals without diabetes [3]. DKD proceeds in stages: (1) an increase in albuminuria (or microalbuminuria, 30 to 300 mg/g urinary albumin); (2) progressing to macroalbuminuria or proteinuria (>300 mg/g); (3) loss of kidney function (GFR < 30 ml/min); and finally (4) requiring renal replacement. Recent evidence suggests a new facet of nephron function, with the proximal tubule playing a part in DKD pathophysiology [4] in addition to having a role as a filtration barrier in glomerular haemodynamics.

DKD development may be primarily determined by proximal tubule injury, which is connected to glomerulus hyperfiltration and glomerular barrier damage via mechanisms modulating albumin excretion and re-uptake [4]. Glomerular hyperfiltration, which occurs early in the course of DKD, is augmented by the hyperglycaemic state in diabetes via increased filtering of glucose. This stimulates the proximal tubule to reabsorb glucose which, coupled with sodium reabsorption, results in vasorelaxation of the afferent artery and increased renal blood flow [5]. Albuminuria is a pivotal biomarker among diabetic individuals who develop DKD, reflecting glomerular and tubular dysfunction [6]. It may also reflect a generalised endothelial dysfunction and is associated with an increased risk of cardiovascular events in diabetic individuals [7, 8].

Family studies suggest that genetic factors explain 16–49% of albuminuria [9]. While several genome-wide association studies (GWASs) of albuminuria have been performed to date, most have focused on identifying common genetic variants (minor allele frequency [MAF] ≥ 5%) for albuminuria [10–12]. Recently, we identified rare coding variants for kidney function (estimated GFR [eGFR]) and development in an exome-wide association study (ExWAS) [13]. Here, we used a similar approach to identify rare (MAF < 1%) or low-frequency (MAF 1–5%) coding variants for albuminuria in 33,985 individuals of European ancestry with (*n* = 15,872) and without (*n* = 18,113) diabetes.

## Methods

### Study populations

The present study comprises a two-stage design: discovery and replication. The discovery set includes five cohorts from Denmark (Inter99, Health2006, Health2008, Vejle Biobank and the Anglo–Danish–Dutch Study of Intensive Treatment In People with Screen Detected Diabetes in Primary Care (Addition)-DK, [Table 1]), with a total of 13,226 participants (3896 with and 9330 without type 2 diabetes), as described previously [14, 15] (electronic supplementary material [ESM] Methods 1.1 Discovery Phase). The first three are population based while the last two are type 2 diabetes case cohorts.

**Table 1:** Clinical characteristics of the individual cohorts: pooled and stratified on diabetes status: discovery and replication stages

Study name	Sample set type	*n*	Women, %	Age, years	ACR^a^ (mg/mmol) or AER^b^ (mg/24 h)	Diabetes, %	Diabetes type	Diabetes duration, years
Stage 1 (discovery)								
Addition-DK^a^	All	2013	47.3	59.6 (7.0)	0.6 (0.2–1.59)^a^	81.6	Type 2	–
	DM	1643	43.9	60.1 (6.8)	0.6 (0.2–1.7)^a^			–
	Non-DM	370	62.4	57.6 (7.4)	0.5 (0.2–1.39)^a^			–
Health2006^a^	All	2658	51.3	49.6 (12.8)	0.57 (0.45–0.9)^a^	0	–	–
Health2010^a^	All	642	55.6	46.6 (8.2)	0.45 (0.34–0.57)^a^	0	–	–
Inter99^a^	All	5971	50.9	46.1 (7.9)	0.34 (0.23–0.57)^a^	5.2	Type 2	–
	DM	311	37.6	51.0 (7.1)	0.45 (0.34–1.13)^a^			–
	Non-DM	5660	51.6	45.9 (7.8)	0.34 (0.23–0.57)^a^			–
Vejle Biobank^c^	DM	1942	38.1	63.4 (8.7)	0.96 (0.54–1.83)^a^	100	Type 2	–
Stage 2 (replication 1): Europeans								
DanFunD^a^	All	7364	47.3	52.1 (13.1)	0.57 (0.57–0.57)^a^	6.3	Type 2	–
	DM	449	43.9	60.4 (8.8)	0.57 (0.57–1.47)^a^			–
	Non-DM	6915	62.4	51.4 (13.3)	0.57 (0.57–0.57)^a^			–
Genesis/Genediab^b^	DM	1249	52.2	42.2 (11.9)	0.11 (0.51–1.36)^a^	100	Type 1	25.6 (10.2)
MDCS^a^	All	2641	56.5	73.0 (5.6)	0.6 (0.4–1.2)^a^	21	Type 2	–
	DM	547	48.3	73.6 (5.4)	0.8 (0.4–2.2)^a^			–
	Non-DM	2094	58.6	72.9 (5.6)	0.6 (0.4–1.1)^a^			–
SUMMIT Consortium (diabetes cohorts)								
Benedict (Phase A and B)^c^	DM	324	32.7	68.1 (7.7)	17.3 (4.6–55.6)^b^	100	Type 2	17.2 (7.1)
Cambridge^b^	DM	245	46.9	23.1 (9.9)	0.78 (0.52–1.71)^a^	100	Type 1	15.7 (6.5)
Eurodiab^b^	DM	680	50.0	42.5 (9.9)	23.9 (22.6–25.3)^b^	100	Type 1	24.7 (8.4)
FinnDiane^b^	DM	2840	50.6	43.9 (11.7)	11.0 (5.0–89.6)^b^	100	Type 1	29.1 (10.0)
GoDarts 1^c^	DM	3530	46.0	67.0 (0.7)	46.1 (38.7–52.6)^a^	100	Type 2	7.5 (6.0)
GoDarts 2^c^	DM	2805	43.0	66.8 (11.8)	50.5 (40.7–59.3)^a^	100	Type 2	7.3 (6.2)
SDR (Type1)^b^	DM	598	43.0	48.6 (13.7)	8.6 (4.3–50.4)^b^	100	Type 1	31.9 (13.3)
SDR (Type2)^c^	DM	1426	41.3	65.6 (10.7)	18.7 (7.2–100.8)^b^	100	Type 2	14.3 (7.6)
Steno Type 2 Diabetes^c^	DM	295	39.3	61.5 (8.1)	50.5 (13.9–893.0)^b^	100	Type 2	15.1 (6.8)
Stage 2 (replication 2): Greenlanders								
Greenlanders^a^ (IHIT + B99)	All	2605 (IHIT = 2519; B99 = 86)	53.7	44.1 (14.5)	0.9 (0.68–1.58)^a^	8.1	–	–

Data for age and diabetes duration are shown as mean ± standard deviation; ACR (mg/mmol)/AER (mg/24 h) are represented as median (interquartile range). For some studies, AER was converted from μg/min to mg/24 h with a multiplication factor of 1.44 (μg/min × 1.44 = mg/24 h). ACR was converted from mg/g to mg/mmol by a multiplication factor of 0.113 (mg/g × 0.113 = mg/mmol)

In sample set type, ‘All’ is all the individuals in the cohort (with and without diabetes). These sets are further stratified based on presence or absence of diabetes. The number of individuals in the phenotyping summary may not match the association summary numbers in actual analyses because genetic information is missing for some individuals

^a^Population**-**based studies

^b^Type 1 diabetes study

^c^Type 2 diabetes study

DM, diabetes mellitus (with); Go-DARTS, Genetics of Diabetes Audit and Research in Tayside Scotland; IHIT, Inuit Health in Transition study; non-DM, without diabetes mellitus; SDR, Scania Diabetes Registry

The replication set includes multiple studies of European descent (*n* = 20,759) involving 11,976 individuals with and 8783 without diabetes (Table 1). These comprise the Danish study of functional disorders (DanFunD) [16], Malmö Diet and Cancer Study (MDCS) [17], Genesis/Genediab [18], Innovative Medicines Initiative – Surrogate markers for Micro- and Macro-vascular hard endpoints for Innovative diabetes Tools (IMI-SUMMIT) Consortia (Europe/UK-based consortia on diabetes studies) [19] and Greenlandic Inuit populations (*n* = 2605) [20]. The DanFunD, MDCS and Greenlandic studies were population based, whereas the Genesis/Genediab studies involved people with type 1 diabetes and the IMI-SUMMIT comprised four type 1 and five type 2 diabetes studies. All studies are described in ESM Methods 1.2 Replication Phase.

None of the studies overlapped with the previous albuminuria GWASs except two replication cohorts within the IMI-SUMMIT Consortia (Finnish Diabetic Nephropathy Study [FinnDiane] and Scania Diabetes Register) that participated in the type 1 diabetes albuminuria GWAS [12].

The present study was conducted in accordance with the Helsinki Declaration and all the participating studies were approved by their respective data protection boards and by the regional scientific ethics committees. Informed consent from all participants was obtained.

### Albuminuria measurements

Albuminuria was diagnosed from a 24 h urine collection (mg/24 h), also called the urinary albumin excretion rate (AER) or from spot urine samples measuring urinary albumin and creatinine concentrations and calculating the urinary albumin/creatinine ratio (ACR in mg/mmol). The summary measures for AER and ACR in the participating cohorts have been described in Table 1 and methods described in ESM Table 1.

### Genotyping and SNP quality control

Genotyping of the discovery stage studies was performed on the Illumina HumanExome BeadChip 12V.1.0 containing 263,894 single nucleotide polymorphisms (SNPs) and including an additional 16,340 custom-typed SNPs from the Danish Exome Sequencing Project as described previously [14, 21] and briefly in ESM Methods 1.3 Danish Exome Sequencing based SNP Selection. Most SNPs were exome based (non-synonymous/coding) gene variants (~90%); thus, we refer to the present association study as an ExWAS. Genotype calling on the discovery set cohorts was performed using the Illumina GenCall plus the zCall algorithm to improve rare variant calling [22]. We excluded SNPs based on: (1) cluster separation score <0.4; (2) Hardy–Weinberg equilibrium *p* < 10^−6^; and (3) call rate <98%. We also excluded individuals with: (1) sex mismatches; (2) genetic duplicates; (3) call rate <95%; and (4) no clustering with the European-ancestry-specific SNPs through a principal component analyses (PCA) approach seeded with ancestry informative markers (AIMs), as described previously [23]. After quality control, a total of 142,397 SNPs remained for a total of 13,226 individuals with complete phenotype and genotype data in the discovery set. Details of the replication cohorts are provided in ESM Table 2. All SNP positions are based on the Genome Reference Consortium Human Build 37 (GRCh37) of dbSNP (https://www.ncbi.nlm.nih.gov/assembly/GCF_000001405.13/).

One replication (MDCS) cohort used Illumina Exome array-based genotyped data, the Greenlandic cohort used Illumina Metabochip-based genotype data [20] while other cohorts had SNP data available from exome-/genome-wide array imputation.

### Statistical analyses

#### Discovery stage

The discovery stage exome-wide association analysis was first performed in each of the five participating studies individually using additive linear regression model and adjusting for sex, age and population sub-structure (principal components; see Project analyses plan in ESM Methods 1.4). Albuminuria measures were natural-log transformed to correct for non-normalised data. The study-specific results were meta-analysed using inverse variance-weighted fixed-effects meta-analysis with weights proportional to the squared standard errors of the effect estimates. The genomic inflation factor (λ) was at acceptable levels both in the individual association analysis (λ_Inter99_ = 1.01, λ_Health2006_ = 1.0, λ_Health2008_ = 1.0, λ_Vejle_ = 0.99, λ_Addition-DK_ = 1.01) and in the combined discovery meta-exome-wide association analysis (λ_discovery_ = 1.0). A *χ*^2^ test for heterogeneity was implemented to estimate the heterogeneity in effect size across the participating studies. The proportion of phenotypic variance (*r*^*2*^ or the coefficient of determination) associated with the top SNP was estimated through a linear regression model (trait ~ SNP + covariates) with the covariates age + sex + principal components (PCs). METAL software [24] was used for the meta-analysis and the R meta package [25] for constructing meta-forest plots. Ancestry-specific linkage disequilibrium (LD) between variants was extracted using the National Institutes of Health (NIH)-based LDlink database [26].

#### Replication stage

SNPs with *p* < 5.0 × 10^−5^ in the discovery meta-analysis were tested for replication in European (*n* = 20,759) and Greenlandic (*n* = 2605) study populations using a similar approach to that used in the discovery analysis. The covariates used for each analysis are given in ESM Table 2. SNPs with imputation quality *r*^2^ < 0.3 were not used for replication (Genesis/Genediab *CUBN* SNP). Following this, a fixed-effects meta-analysis using either inverse variance weighting (wherever possible) or a weighted sum of *z* scores was performed. For the IMI-SUMMIT Consortium, no effect sizes or standard errors were available in the summary results. Hence, we performed the replication meta-analysis for the *KCNK5* rs10947789 and *LMX1B* rs140177498 SNPs, for which the IMI-SUMMIT Consortium contributed data, by using the weighted sum of *z* scores. Replication meta-analysis with *p*_Bonferroni_ = 0.017 (three SNPs) was considered significant.

#### Combined meta-analysis

The combined meta-analysis was performed with all individuals of European ancestry (Eur) followed by pooling with Greenlandic data (Eur–GL).

Any SNP with *p*_replication_ < 0.017 and *p*_meta_Eur/Eur–GL_ < 5.0 × 10^−8^ was considered overall significant.

#### Diabetes-stratified analysis

Diabetes-stratified SNP–albuminuria association (for index SNPs) was assessed in the discovery set (with diabetes, *n* = 3896; non-diabetes, *n* = 9330) after pooling individual genotype data on all participants and verified through an interaction regression model (trait ~ SNP + diabetes_status + [SNP×diabetes_status] + age + sex + cohort + PC_1_–PC_4_).

#### Conditional analyses

Conditional analyses for novel SNPs identified in known loci (and/or in low LD, *r*^2^ < 0.01) were performed to determine if the signal was independent. The following linear model was used: trait ~ top identified SNP + secondary known SNP + sex + age + PCs. If the top SNP retained the association estimates and *p* value it was considered an independent signal.

#### Gene-aggregate tests

Gene-based multi-marker association testing for rare and common exonic and intragenic variants (after removing monomorphic variants) was performed using the Meta Analysis for SNP–set (sequence) kernel association test (MetaSKAT) R package [27]. SNPs were filtered based on their annotation status in the Genome Variation Server (GVS: SeattleSeq Annotation 138/hg19), where SNPs belonging to the following categories were taken forward to the gene-based analyses: missense, missense-near-splice, splice-3, splice-5, coding-synonymous, stop-gained, stop-gained-near-splice, stop-lost, stop-lost-near-splice, untranslated region (UTR)-3, UTR-5 and intronic. Intergenic variants or singletons that did not fall under the annotated gene sets were removed, with 18,026 valid gene sets remaining.

At the study-specific level, the gene-based analyses were performed against the null model (using SKAT-O method) [27], accounting for sex and ten PCs, generating SKAT objects individually for each cohort with available genotyped data (discovery + MDCS study, six studies) which were then meta-analysed in a single stage to incorporate maximum power for testing the rare variants cumulatively.

The meta-analysis of the summary-level score statistics was run using the Hom-Meta-SKAT-O ‘optimal’ method, which assumes that different studies share the same causal variant, weighting them equally. A Bonferroni threshold (*p*_adjusted_ < 2.7 × 10^−6^) based on 18,026 annotated gene sets was used.

#### Additional SNP–trait associations

We examined the associations of the exome-wide significant index SNPs with type 2 diabetes risk and kidney function (eGFR) in the discovery set. The Pheno Scanner database (www.phenoscanner.medschl.cam.ac.uk/phenoscanner), comprising publicly available results for GWASs and expression quantitative trait loci (eQTL) studies [28], as well as the GWAS summary results for type 2 diabetes from the DIAbetes Genetics Replication And Meta-analysis (DIAGRAM) consortium, were also accessed to mine known SNP–trait associations for the index SNPs.

#### SNP functionality prediction

We used the Combined Annotation Dependent Depletion (CADD) database to predict variants as per their functional categories (deleterious, disease causal, pathogenicity) through integrating multiple annotations into one metric [29]. A scaled CADD or C score/PHRED [−10 × log_10_(rank/total)] is a ranking for a variant relative to all possible substitutions of the human genome (8.6 × 10^9^).

## Results

### Albuminuria ExWAS

#### Discovery stage

**Fig. 1:**
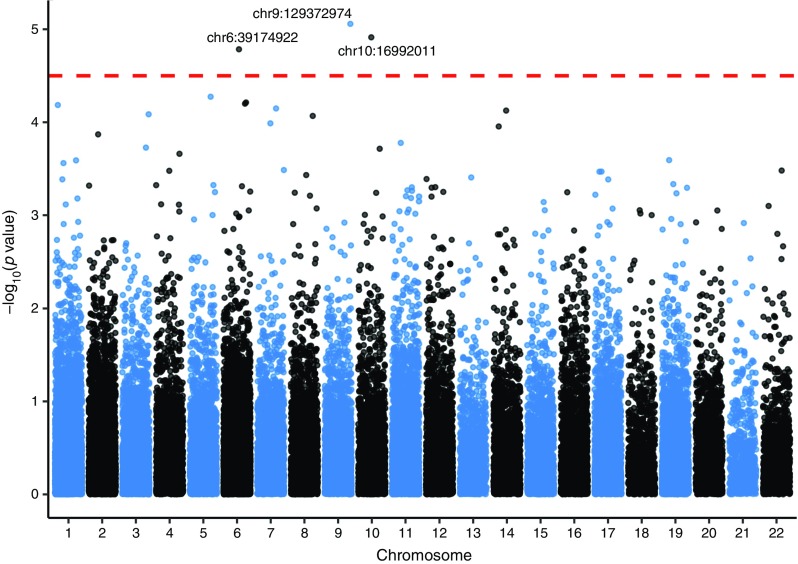
Manhattan Plot for the discovery stage meta-ExWAS at *p*_discovery_ ***<*** 5.0 × 10^−5^. The *x*-axis shows the chromosome number and the *y*-axis shows −log_10_(*p* values) for the SNP–albuminuria association. Index SNPs are named as chromosome number:position (GRCh37 of dbSNP): chr10:16992011, *CUBN* rs141640975; chr6:39174922, *KCNK5* rs10947789; chr9:129372974, *LMX1B* rs140177498. Chr, chromosome

**Fig. 2:**
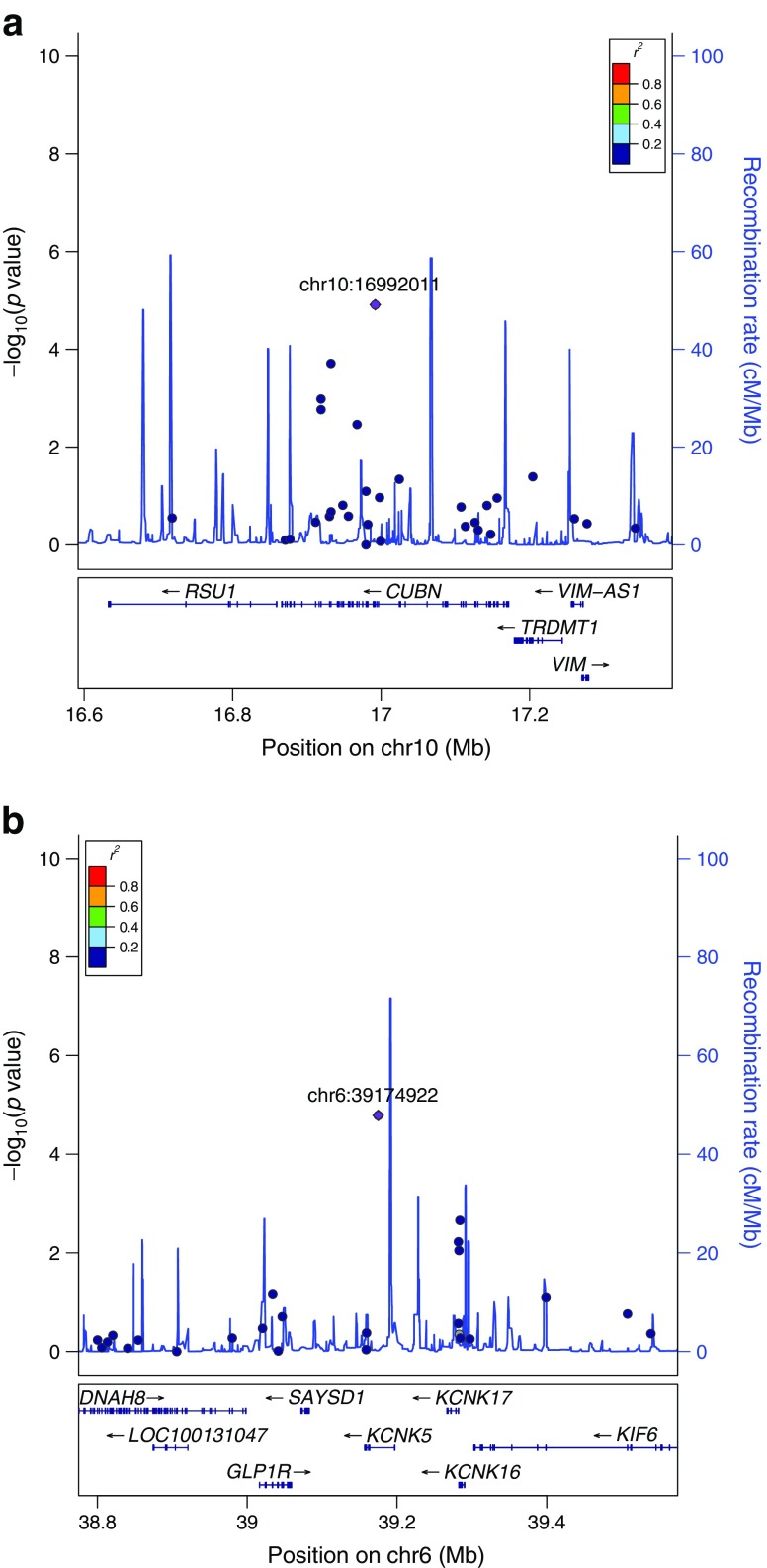
(**a**) Regional plot for *CUBN* rs141640975 on chromosome 10 (for space reasons, the *ST8SIA6* gene is omitted from the right of the key below). (**b**) Regional plot for *KCNK5* rs10947789 on chromosome 6. Index SNPs are named as chromosome number:position (GRCh37 of dbSNP): chr10:16992011, *CUBN* rs141640975; chr6:39174922, *KCNK5* rs10947789. Chr, chromosome. The diamond (shown in purple) is the most significant SNP in the region (index SNP), based on *p* value; all other SNPs are shown as circles. The colours represent the degree of LD with the index SNP (see *r*^2^ values in the key); grey represents unavailability of LD data for a SNP. For additional documentation please see http://locuszoom.sph.umich.edu//

**Fig. 3:**
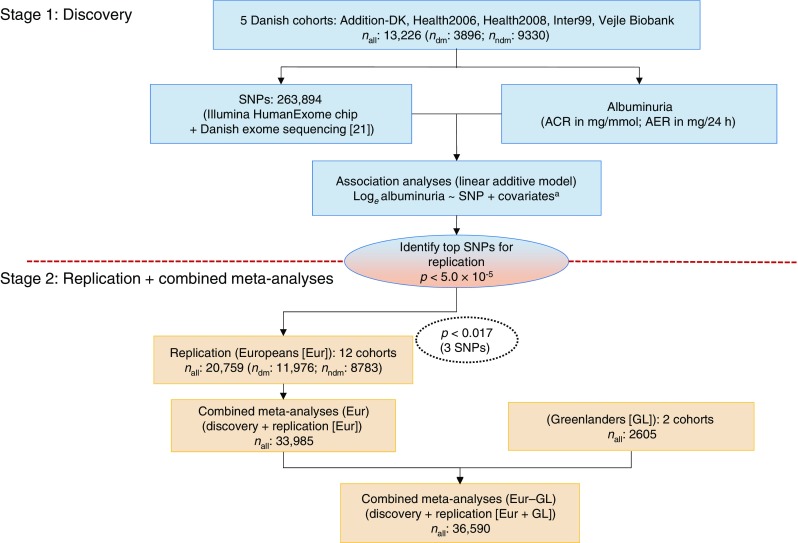
Study design overview. dm, with diabetes; ndm, without diabetes. ^a^covariates: age, sex, PCs

In the discovery stage meta-analysis, three independent SNPs, including two rare variants (MAF <1%) in *CUBN* and *LMX1B* and a common variant in *KCNK5*, attained *p*_discovery_ < 5.0 × 10^−5^ (Table 2). The Manhattan, QQ and LocusZoom (http://locuszoom.sph.umich.edu//) plots for the discovery meta-analysis are shown in Fig. 1, ESM Fig. 1 and Figs 2 and 3 and genotype-stratified study characteristics are given in ESM Table 3.

**Table 2:** Associations for the top SNPs from ExWAS discovery, replication and combined meta-analyses, including Europeans and Europeans + Greenlanders, for albuminuria

SNP characteristics					Discovery *n =* 13,226				Replication *n =* 20,759				Combined (Eur) *n* = 33,985		Combined (Eur–GL) *n* = 36,590		
SNP (rsID)^a^	Gene	Chr (BP)	Anno	EA/OA	EAF (%)	β	SE	*p*_discovery_	EAF (%)	*n*	β/DOE	*p*_replication_	*n*	*p*_Eur_	*n*	*p*_Eur–GL_	*p*_het_
rs141640975	*CUBN*	10 (16992011)	missense (A1690V)	A/G	0. 8	0.266	0.061	1.2 × 10^−5^	0. 8	9742	0.279	2.9 × 10^−7^	22,866	1.3 × 10^−11^	–	–	–
rs10947789	*KCNK5*	6 (39174922)	Intronic	C/T	23	0.054	0.012	1.6 × 10^−5^	24	20,759	+	0.03	33,985	1.5 × 10^−5^	36,590	9.1 × 10^−6^	0.84
rs140177498	*LMX1B*	9 (129372974)	Upstream	T/C	0.9	0.260	0.058	8.7 × 10^−6^	1.1	13,233	+	0.43	–	–	–	–	–

SNPs with *p*_*Eur/Eur–GL*_ < 5.0 × 10^−8^ are novel

Discovery set is based on up to 13,226 individuals (3896 with and 9330 without diabetes)

EAF (Greenlanders, rs10947789) = 45%

EAF values are in %

β values correspond to natural log transformed albuminuria levels (ACR in mg/mmol or AER in mg/24 h)

*p* values are for association

^a^SNPs were selected for replication based on *p*_discovery_ < 5.0 × 10^−5^

Anno, SNP annotation; BP, SNP position in base pairs from the Genome Reference Consortium Human Build 37 (GRCh37) in dbSNP; Chr, chromosome; DOE, direction of effect corresponding to the association summary meta-analysis based on *z* scores for the respective SNP; EA/OA, effect allele/other allele; ID, identity

The *CUBN* rs141640975 is a rare (MAF 0. 83%) missense (A1690V) SNP with the A allele associated with increased albuminuria (β = 0.25; *p*_discovery_ = 1.2 × 10^−5^). Genotype vs mean albuminuria levels for the *CUBN* rs141640975 are shown in ESM Fig. 2 (levels for *KCNK5* and *LMX1B* SNPs are shown in ESM Figs 3, 4, respectively), whereas a forest plot depicting the effect estimates for each study in the discovery meta-analysis is shown in ESM Fig. 5. The *KCNK5* (which encodes potassium two-pore domain channel subfamily K member 5) common (MAF 23%) intronic SNP rs10947789 C allele and the *LMX1B* (encoding LIM homeobox transcription factor 1 β) rare (MAF 0.89%) intronic SNP rs140177498 T allele associated with increased albuminuria (s10947789, β = 0.05, *p*_discovery_ = 1.6 × 10^−5^; ESM Fig. 6; rs140177498, β = 0.26, *p*_discovery_ = 8.7 × 10^−6^; ESM Fig. 7). Systolic BP-adjusted associations are shown in ESM Table 4.

#### Replication stage

Replication of the three SNPs was sought in up to 20,759 individuals (with diabetes, *n* = 11,976; without diabetes, *n* = 8783).

The *CUBN* rs141640975 replicated strongly (*n*_replication_*CUBN*_ = 9742; *p*_replication_ = 2.8 × 10^−7^), while the *KCNK5* rs10947789 was close (*n*_replication_*KCNK5*_ = 20,757; *p*_replication_ = 0.03). The *LMX1B* rs140177498 did not replicate (*n*_replication_*LMX1B*_ = 13,233; *p*_replication_ = 0.43) (Table 2**)** and was not analysed further, whereas we continued to look into *KCNK5*.

#### Combined meta-analysis

The combined meta-analysis (discovery + replication) comprised 33,985 European individuals while the combined European–Greenland group comprised 36,590 individuals (Table 2). Only the *KCNK5* SNP was available in the Greenlandic Illumina Metabochip data, and here the *KCNK5* rs10947789 C allele had a frequency of 45%.

The C*UBN* rs141640975 remained significant overall after the Eur meta-analysis (*p*_meta_Eur_ = 1.3 × 10^−11^) while the *KCNK5* SNP was non-significant with *p*_meta_Eur–GL_ = 9.1 × 10^−6^ in the Eur–GL meta-analysis (Table 2). The overall study design for single SNP testing is shown in Fig. 3.

### Diabetes-stratified association

**Fig. 4:**
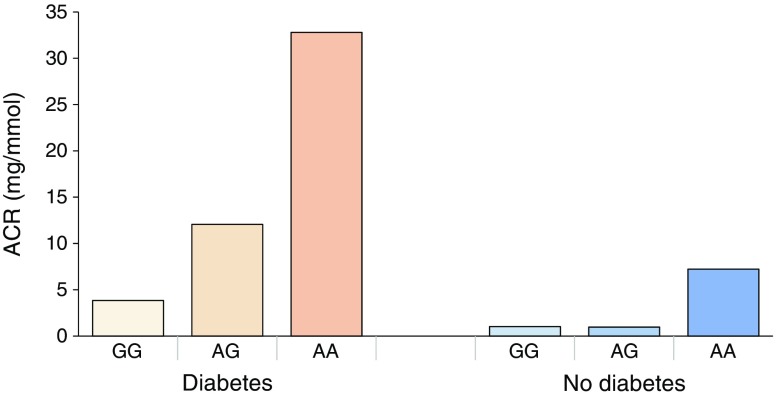
ACR levels stratified by *CUBN* rs141640975 genotype (GG, AG and AA) and type 2 diabetes status in the discovery cohorts. Diabetes, with GG *n* = 3806, with GA *n* = 66, with AA *n* = 1; no diabetes, with GG *n* = 9270, with GA *n* = 152, with AA *n* = 1

The effect estimates of *CUBN* rs141640975 were more than threefold higher in the type 2 diabetes group (β = 0.69; *p* = 2.0 × 10^−5^; ESM Fig. 8) in comparison with the non-diabetes group (β = 0.20; *p* = 0.002; ESM Fig. 9) with a significant interaction based on diabetes status (*p*_interaction_ = 7.0 × 10^−4^) (Fig. 4). No significant interaction was observed for the *KCNK5* SNP (*p*_interaction_ = 0.077). Replication studies were not included in this analysis because individual-level genotype data were not available for all cohorts.

### Conditional analyses

Conditional analysis for the identified C*UBN* rare variant rs141640975 (GRCh37.p13 position: 16,992,011, MAF 0.83%) was carried out for the known *CUBN* common SNP rs1801239 (GRCh37.p13 position 16,919,052, MAF 10%), which occurs at a distance of ~73 kbp (LD *r*^2^ = 0.0002, D′ = 1.0). The effect estimates for the novel variant rs141640975 did not change before (*p*_rs141640975_ = 8.8 × 10^−7^, β = 0.33) or after (*p*_rs141640975_condition_ = 8.5 × 10^−7^, β = 0.33) conditioning with the known *CUBN* SNP rs1801239 (*p*_*rs1801239*_ = 0.0002, β = 0.05), which is also in LD with another reported SNP rs17343073 (LD *r*^2^ = 0.92, D′ = 1.0). Similar conditioning for other known *CUBN* common SNPs (rs6602163/rs10795433) was performed in the same or proxy SNP (LD, *r*^2^ ≥ 0.6, ESM Table 5).

### Power calculations

We performed power calculations for the single SNP analyses using the Genetic Association Study (GAS) power calculator for GWAS [30]. For the rare variant (rs141640975), assuming an additive disease model, α = 5 × 10^−8^, *n*~33,000, MAF~1% and relative risk ~ 1.5, with disease prevalence = 0.15, the expected power was 81%. For the common variant (rs10947789) α = 5 × 10^−8^, *n*~33,000, MAF ~ 23% and relative risk ~ 1.1, the expected power was 62%.

### Gene-aggregate tests

Applying the Hom-O-SKAT Meta (weighted) optimal test from six studies comprising a total of 15,867 individuals, we identified three genes *HES1* (*p* = 3.7 × 10^−9^) in chromosome 3, *CDC73* (*p* = 6.4 × 10^−9^) in chromosome 1 and *GRM5* (*p* = 1.6 × 10^−6^) in chromosome 11 (Table 3) surviving Bonferroni correction (*p*_Bonferroni_ *<* 2.7 × 10^−6^, 18,026 gene sets). Association results for individual SNPs included in the gene-aggregate tests for these three genes are shown in ESM Table 6, whereas the overall analysis design is shown in ESM Fig. 10.

**Table 3:** Genes associated with albuminuria through gene-aggregate tests

Gene	Chr	*p* value	No. SNPs/set	Full gene name	Alternative gene symbols	Gene ID (NCBI) 21 Dec 2017
*HES1*	3q29	3.7 × 10^−9^	2	Hes family bHLH transcription factor 1	*HHL*; *HRY*; *HES-1*; *bHLHb39*	3280
*CDC73*	1q312	6.4 × 10^−9^	2	Cell division cycle 73	*HYX*; *FIHP*; *HPTJT*; *HRPT1*; *HRPT*2; *C1orf28*	79,577
*GRM5*	11q142–q143	1.6 × 10^−6^	7	Glutamate metabotropic receptor 5	*mGlu5*; *GPRC1E*; *MGLUR5*; *PPP1R86*	2915

All results significant at *p* < 2.7 × 10^−6^, after correction for (Bonferroni’s) multiple testing and are based on six study cohorts (discovery set + MDCS) Inclusions for gene-aggregate tests are based on annotated exonic or intragenic SNPs, totalling *n* = 18,026 gene sets examined

Detailed results for the identified genes are available in ESM Table 6

The SKAT-cohort function was used to run each cohort, adjusting for age, sex, PCs + study-specific covariates. Following this, the Meta SKAT function and Hom-O-SKAT Meta (weighted) model was used to meta-analyse individual cohort data. The rest of the replication studies did not have data/resources available to run these analyses and therefore could not be incorporated. A brief overview of the gene-aggregate testing is presented in ESM Fig. 10

Chr, chromosome; ID, identity; no., number

### Additional SNP–trait associations

The *CUBN* rs141640975 A allele was not associated with type 2 diabetes risk in the discovery set (*p* > 0.05) or in the DIAGRAM Consortium summary results (*p* > 0.05 in models unadjusted and adjusted for BMI; ESM Table 7). However, the A allele showed a nominally significant association with increased eGFR (*p* = 0.04, β = 0.026) within the discovery set (ESM Table 7).

The publicly available report suggests that the *CUBN* rare SNP A allele is associated with reduced serum creatinine levels in a blood-based metabolomics study (*p* = 0.014, β = −0.28).

The *KCNK5* rs10947789 C allele associated with increased urinary ACR (β = 0.025, *p* = 5.6 × 10^−4^) in CKD Genetics Consortia-based research. Other *KCNK5*-associated traits include myocardial infarction (CARDIoGRAMplusC4D Consortia, β = −0.059, *p* = 1.4 × 10^−6^), visual refractive error (β = 0.11, *p* = 0.003) and birthweight (Early Growth Genetics [EGG] Consortium, β = 0.025, *p* < 0.007) in addition to *KCNK5* being a GWAS locus for coronary artery disease.

eQTL data suggest rs10947789 C allele-specific gene expression associations within the adrenal gland (β = 0.51, *p* = 2.5 × 10^−5^), subcutaneous adipose tissue (β = 0.34, *p*: 7.2 × 10^−5^), lymphoblastoid cell line (β = 0.03, *p* = 0.0019) and tibial nerve (β = 0.15, *p* = 0.002).

Detailed SNP–trait associations with references are documented in ESM Tables 8–10.

### SNP functionality prediction

The *CUBN* missense SNP rs141640975 was described as functionally ‘deleterious’ with SIFT, ‘probably damaging’ with PolyPhen and had a CADD (scaled C score) of 24.5.

## Discussion

In the combined meta-ExWAS of 33,985 Europeans (five discovery and 12 replication cohorts), we identified one novel *CUBN* variant associated with albuminuria levels and exerting >3.5-fold increased effects among individuals with type 2 diabetes compared with non-diabetic individuals.

Although *CUBN* is a known locus for albuminuria, the identified rare missense variant shows independent effects (with respect to known SNPs in *CUBN*) that are stronger within the diabetes vs the non-diabetes group (*p*_interaction_ = 7.0 × 10^−4^). This rare variant explains up to 6.4% of variance per rare allele (in a model adjusted for age and sex) in albuminuria levels (natural log transformed). Also, the gene-based tests identify three additional genes (*HES1*, *CDC73* and *GRM5*) that associate with albuminuria (*p*_Bonferroni_ < 2.7 × 10^−6^) in a meta-analysis comprising six Scandinavian cohorts.

There have been a few albuminuria GWASs in the past decade [10–12, 31], all exploring the common genetic variants (MAF>5%), but in the current study we examined low-frequency and rare variants, particularly from the coding region (exome) of the genome.

While common variants in *CUBN* have been previously reported to associate with albuminuria in individuals of European, African and Hispanic ancestry [10, 32], the rare missense (A1690V) SNP rs141640975 in *CUBN* that we identified is not in LD with the recently reported *CUBN* SNPs rs1801239 (*r*^2^_LD_ = 0.0002, D′_LD_ = 1.0) [10] and rs6602163 (*r*^2^_LD_ = 0.0008, D′_LD_ = 1.0) [11] for European ancestry. This is confirmed in the current study through conditional analyses (rs1801239/rs17343073, *p*_conditional_ = 8.5 × 10^−7^ and rs6602163/rs10795433, *p*_conditional_ = 4.9 × 10^−7^), with the minor allele associated with increased albuminuria.

A strong interaction between diabetes status and *CUBN* missense rs141640975 observed with respect to albuminuria in the current study suggests potential clinical implications. Cubilin, encoded by the *CUBN* gene*,* is expressed in the apical brush border of proximal renal tubule cells and forms a complex with megalin protein to promote albumin re-uptake [10, 33]. An important mechanism underlying DKD is tubulointerstitial damage involving the proximal tubule [4]. A diabetic or hyperglycaemic state alters the tubular function by augmenting glomerular hyperfiltration [4, 5], while the proximal tubule may determine the level of glomerular hyperfiltration through glucose reabsorption [34]. Excess albumin in the urine may be a consequence of defective tubular reabsorption in diabetic individuals, which could be protective against DKD by reducing the reabsorption of glucose. A recent study on diabetic mice identified a lower expression of cubilin (part of the endocytic machinery) in the renal cortex and proximal tubule and a correlation between tubular endocytosis dysfunction and higher urinary excretion of albumin, transferrin and total protein [35], highlighting the critical role of cubilin and the proximal tubule in the diabetic state.

As cubilin protein is a co-receptor not only for tubular resorption but also for the intestinal vitamin B_12_–intrinsic factor complex, *CUBN* mutations lead to a hereditary form of megaloblastic anaemia (or Imerslund–Gräsbeck syndrome) characterised by tubular proteinuria and vitamin B_12_ malabsorption [36, 37]. Moreover, a recent exome-sequencing study revealed a homozygous frameshift mutation in *CUBN* associated with the only cause of proteinuria in affected family members [37]. Despite *CUBN* being a disease gene, recent exome-sequencing studies and related reference databases (ExAC [38]) have shown that damaging variants are rather frequent in ‘non-diseased’ populations and are thus well tolerated by humans [38, 39]. On this basis, it was recently hypothesised that the tubular proteinuria caused by cubilin deficiency could actually be protective against tubular overload, seen, for example, in nephrotic syndrome or even DKD [39]. As *CUBN* rs141640975 has been associated with lower serum creatinine (*p* = 0.014) in a recent meta-GWAS of circulating metabolites [40] and causes albuminuria also in the general population group, our study supports the idea that functional variations in *CUBN* might not be damaging but instead protective. The CADD/SIFT database testing of functionality of the non-synonymous SNPs that result in amino acid changes suggests *CUBN* rs141640975, with a high C score and deleterious nature, to be of functional importance. Indeed, we found that the albuminuria-increasing rs141640975 A allele was associated with more efficient kidney function in the discovery set (*p* = 0.04), suggesting it may be protective against DKD. However, further functional and validation studies are required to shed light on the potential protective effect of this *CUBN* variant.

Although no other SNP was replicated in the single SNP analyses, the *KCNK5* gene was close to the replication threshold (*p* = 0.03), though it did not reach the GWAS threshold in the combined Eur–GL meta-analysis (*p =* 9.1 × 10^−6^). *KCNK5* is a known coronary artery disease GWAS locus [41], encoding the potassium two-pore domain channel subfamily K member 5 protein, which is mainly expressed in the cortical distal tubules and collecting ducts of the kidney [42]. This protein is highly sensitive to pH, and functional inactivation may lead to renal acidosis [43]. Data mining revealed the rs10947789 minor allele (C) to be associated with increased albuminuria in the CKDGen Consortia [11], supporting our findings. eQTL-based look-ups indicate rs10947789 associated strongly with *KCNK5* expression in the adrenal gland and subcutaneous adipose tissue (*p* < 8.0 × 10^−5^), suggesting a functional role in the kidneys. Albeit *KCNK5* rs10947789 has promising roles with respect to the cardio–renal axis, the power calculation in the current study (62%) suggests that the variant has smaller effects; a larger sample size may be required to validate its association with albuminuria.

The *HES1* gene identified through the gene-aggregate tests is a transcription factor ubiquitously expressed in most organs, including the kidneys; it has been documented to be involved in Notch signalling pathways that play a role in renal fibrosis [44], glomerulosclerosis [45] and other forms of kidney disease [44, 46]. The *CDC73* gene is a tumour suppressor gene, mutations in which have been associated with hyperparathyroidism–jaw tumour syndrome and familial hyperparathyroidism [47]. Albuminuria is associated with hyperparathyroidism, which is a complication of CKD [48], and the present findings thus suggest a plausible link between the two.

*GRM5* encodes glutamate metabotropic receptor 5, which is a G protein-coupled receptor involved in second messenger signalling. Variants in the metabotropic glutamate receptor group I pathway, including *GRM1* and *GRM5*, were enriched in the pathway analysis of a recent albuminuria GWAS among people with type 1 diabetes from the FinnDiane study [12] (no individuals overlapped with the current gene-aggregate meta-analysis). *GRM5* is also expressed in podocytes and is associated with podocyte apoptosis in animals [49] and pharmacological effects in humans [50].

Albeit the strength of the current study is the identification and validation of a rare variant with optimal power, more statistical power could have improved *KCNK5* validation. Also, testing type 2 diabetes individuals in the discovery stage and including type 1 diabetes cohorts in the replication stage may have introduced some heterogeneity. However, our main finding, the association of the *CUBN* rare variant with albuminuria, remains unaffected as the meta-analysis for this variant only included type 2 diabetes cohorts because of the lack of genotyping or poor imputation quality in type 1 diabetes replication cohorts. Furthermore, the *KCNK5* SNP showed the same direction of effect in both type 1 and type 2 diabetes cohorts, and there was no significant difference in the association of *KCNK5* with albuminuria between these cohorts (*p* for heterogeneity [*p*_het_] > 0.05). For the gene-aggregate testing we used six cohorts (having genotype data) in a single-stage meta-analysis to ensure quality and maximise statistical power for rare variant analysis.

In summary, we identified a rare coding *CUBN* variant implicated in elevated albuminuria levels, especially in individuals with type 2 diabetes. Further, we identified additional novel genes associated with albuminuria through an alternative gene-aggregate approach among Europeans. Our findings provide fresh insights into the genetic architecture of albuminuria and highlight new targets, genes and pathways for the prevention and treatment of DKD.

## Electronic supplementary material

ESM (PDF 1.08 MB)

## Data Availability

All data generated or analysed during this study are included in this published article (and the electronic supplementary material). The summary data for the associations of the index SNP with other traits and with type 2 diabetes that support the findings of this study are available from the PhenoScanner and DIAGRAM Consortium websites (www.phenoscanner.medschl.cam.ac.uk and www.diagram-consortium.org/downloads.html). Additional data are available on request from the authors (T. S. Ahluwalia and/or T. Hansen).

## Abbreviations

ACR: Urinary albumin/creatinine ratio
Addition: Anglo–Danish–Dutch Study of Intensive Treatment In People with Screen Detected Diabetes in Primary Care
AER: Urinary albumin excretion rate
CADD: Combined Annotation Dependent Depletion
CKD: Chronic kidney disease
DanFunD: Danish study of functional disorders
DIAGRAM: DIAbetes Genetics Replication And Meta-analysis
DKD: Diabetic kidney disease
EAF: Effect allele frequency
Eur: All individuals of European ancestry (in combined meta-analysis)
eQTL: Expression quantitative trait loci
Eur–GL: Eur pooled with Greenlandic data (in combined meta-analysis)
ExWAS: Exome-wide association study
FinnDiane: Finnish Diabetic Nephropathy Study
GWAS: Genome-wide association study
IMI-SUMMIT: Innovative Medicines Initiative – Surrogate markers for Micro- and Macro-vascular hard endpoints for Innovative diabetes Tools
LD: Linkage disequilibrium
MAF: Minor allele frequency
MDCS: Malmö Diet and Cancer Study
PCs: Principal components
*p*_het_: *p* for heterogeneity
SKAT: Set (sequence) kernel association test
SNP: Single nucleotide polymorphism
