# Supplementary material for: A novel rare CUBN variant and three additional genes identified in Europeans with and without diabetes: results from an exome-wide association study of albuminuria
Source: Diabetologia. 2018 Dec 13;62(2):292–305. doi: 10.1007/s00125-018-4783-z (PMC6323095; doi:10.1007/s00125-018-4783-z)
Supplement: Supplementary file 1 — (PDF 1.08 MB) [file 125_2018_4783_MOESM1_ESM.pdf]

Supplemental Material (Text and Figures)

A novel rare *CUBN* variant and three additional genes identified in Europeans with and without diabetes: results from an exome-wide association study for albuminuria

I. Electronic Supplementary Material (ESM) Methods

1. Study Description
   1.1 Discovery Phase
      - ADDITION-DK
      - Health2006
      - Health2008
      - Inter99 Study
      - Vejle Biobank

   1.2 Replication Phase
      - The Malmo Diet and Cancer Study (MDCS)
      - Genesis/Gendiab
      - Danfund
      - SUrrogate markers for Micro- and Macro-vascular hard endpoints for Innovative diabetes Tools (SUMMIT Consortia, 9 Studies)
        EURODIAB Family Study
        Finnish Diabetic Nephropathy Study (FinnDiane)
        Cambridge
        Scannia Diabetes Register: Type 1 (SDR1)
        Scannia Diabetes Register: Type 2 (SDR2)
        BENEDICT
        STENO Type 2 Diabetes
        Genetics of Diabetes Audit Research Tayside Scotland 1: Type 2(GoDARTS1)
        Genetics of Diabetes Audit Research Tayside Scotland 2: Type 2(GoDARTS2)
      - Greenlanders

   1.3 Danish Exome Sequencing based SNP selection
   1.4 Project Analyses Plan

II. ESM Tables

ESM Table #	Contents
**1.**	Study Albuminuria measurement Methods
**2.**	Study Genotyping Details
**3.**	Study characteristics stratified by lead (discovery) SNP genotypes
**4.**	SNP-albuminuria associations adjusted for Systolic Blood Pressure
**5.**	Conditional analyses of the *CUBN* rs141640975
**6.**	Detailed results for the genes identified in the gene aggregate tests
**7.**	Association of *CUBN* rs141640975 with type 2 diabetes and kidney function (eGFR)
**8.**	Known GWAS SNP-trait associations for *KCNK5* rs10947789
**9.**	Known eQTL based associations for *KCNK5* rs10947789
**10.**	Known metabolite-SNP associations for *KCNK5* rs10947789 & *CUBN* rs141640975

III. ESM Figures

ESM Figure 1. Quantile-quantile (QQ) plot for the discovery stage albuminuria ExWAS
ESM Figure 2. Bar plot of the *CUBN* rs141640975 genotypes versus mean albuminuria levels (mg/g) in the discovery set.
ESM Figure 3. Bar plot of the *KCNK5* rs10947789 genotypes versus mean albuminuria levels (mg/g) in the discovery set.
ESM Figure 4. Bar plot of the *LMX1B* rs140177498 genotypes versus mean albuminuria levels (mg/g) in the discovery set.
ESM Figure 5. Forest plot of the discovery set meta-analysis for the *CUBN* SNP in pooled group
ESM Figure 6. Forest plot of the discovery set meta-analysis for the *KCNK5* SNP in pooled group
ESM Figure 7. Forest plot of the discovery set meta-analysis for the *LMX1B* SNP in pooled group
ESM Figure 8. Forest plot of the discovery set meta-analysis for the *CUBN* in diabetes group (DM)
ESM Figure 9. Forest plot of the discovery set meta-analysis for the *CUBN* SNP in non-diabetes group (NDM)
ESM Figure 10. Gene aggregate testing: Overall analyses design

IV. ESM Tables (Study group members)

ESM Table #	Contents
**11.**	Study Acknowledgement and Funding Statement
**12.**	IMI-SUMMIT Consortia participants list

I. ESM Methods

1. Study Description

Study Populations

Description of the Danish study samples applied in primary association analysis (discovery phase). Clinical and biochemical characteristics for all study sample groups are shown in Main Table 1. All individuals participating in the discovery studies were of Danish nationality. Informed written consent was obtained from all study participants. The studies were conducted in accordance with the Declaration of Helsinki II and were approved by the local Ethical Committees.

ESM Methods 1.1 Discovery Phase

ADDITION DENMARK STUDY [1, 2]

Addition-DK is the Danish arm of the ADDITION-Europe Study (Anglo–Danish–Dutch Study of Intensive Treatment in People with Screen- Detected Diabetes in Primary Care), which is an intervention study based on screening for individuals with high-risk of type 2 diabetes. The sampling is performed at the Department of General Practice, University of Aarhus, Denmark (ClinicalTrials.gov ID-no: NCT00237549). A total of 8,662 individuals from the initial screening cohort had DNA available.

A total of 2,013 individuals with genotype and phenotype data participated in the current study where 1,643 were diabetes cases (screen-detected and untreated type 2 diabetes) while 370 were without diabetes.

HEALTH-2006 [3]

Health2006 is a general population-based cohort, designed to investigate lifestyle-related chronic diseases including diabetes and coronary heart disease. The participants were selected randomly from the South western part of greater Copenhagen area with age group ranging 18-69 years and a participation rate of 44.7% (N=3,471 individuals participating). Health2006 was conducted at the Research Centre for Prevention and Health in Glostrup, Denmark.

In the current study 2,658 individuals with genotype and phenotype data and with no diabetes participated.

HEALTH-2008 [4]

Health2008 is a general population-based cohort, designed to investigate lifestyle-related chronic diseases including diabetes and coronary heart disease. The participants were selected randomly from the South Western part of greater Copenhagen area with age group ranging 30–60 years and a participation rate of 35.8% (N=795 individuals participating). Health2008 was conducted at the Research Centre for Prevention and Health in Glostrup, Denmark.

In the current study 642 individuals with genotype and phenotype data and no diabetes, participated.

INTER99 [5, 6]

The Inter99 cohort is a randomized, non-pharmacological intervention study for the prevention of ischaemic heart disease, conducted on 6,784 randomly ascertained participants aged 30 to 60 years at the Research Centre for Prevention and Health in Glostrup, Denmark (ClinicalTrials.gov: NCT00289237). Detailed characteristics of Inter99 have been published previously.

In the current study 5,971 individuals with genotype and phenotype data participated. 311 had diabetes whereas 5,660 had no diabetes (normal glucose tolerance).

VEJLE BIOBANK [7]

Vejle Biobank situated at Vejle Hospital comprises of clinical-onset type 2 diabetes cases and non-diabetic control individuals with matching age and sex distribution that have been examined during a three year period. Control individuals were confirmed of non-diabetic status when they self-reported and also through a fasting plasma glucose test fulfilling WHO 1999 criteria.
In the current study 1,942 type 2 diabetes cases with genotype and phenotype data participated.

ESM Methods 1.2 Replication Phase

DANFUND [8]

The DanFunD study was initiated at the Research Centre for Prevention and Health, Glostrup, Denmark, as a random sample of the general adult population. The cohort comprises a total of 9,656 individuals aged 18–76 years, born in Denmark and living in the Western part of the greater Copenhagen. The cohort consist of DanFunD part one and DanFunD part two. In the current study, we use the DanFunD part two data. The DanFunD part two was carried out from 2012 to 2015. A total of 7,493 individuals aged 18–72 years participated.

Written informed consent was obtained from all participants, and the study was approved by the Ethical Committee of Copenhagen County (Ethics Committee: KA-2006-0011, H-3-2011-081, and H-3-2012- 0015) and the Danish Data Protection Agency. For more information, please contact DanFunD administrative and scientific officer Thomas Dantoft or visit the webpage at http://www.danfund.org.

GENESIS/GENEDIAB [9, 10]

This is a French study comprising individuals of European ethnicity with type 1 diabetes and have been called GENEDIAB (genes nephropathy and diabetes) and GENESIS (genes nephropathy and sib pair study) based on some sub classifications (diabetes duration).

All type1diabetes patients attending diabetes clinics were invited to be included in the GENEDIAB study, provided they had severe diabetic retinopathy (proliferative or severe non-proliferative), regardless of their nephropathy status. All type 1 diabetes patients with retinopathy and diabetes duration longer than 15 years were included in the GENESIS study.

Diabetes classification was made using American Diabetes Association diagnostic criteria, as defined in 1997. Type 1 diabetes was defined as age at diabetes onset before 35 years. 1,249 individuals with albuminuria measures and genotype data participated in the current study.

The Malmo Diet and Cancer Study (MDCS) [11, 12]

MDCS is a population-based cohort study in the city of Malmö, Sweden. All women and men (n=74,138) born 1923-1950, and 1923-1945, respectively, were invited to participate in the baseline examination between 1991 and 1996. Mental incapacity and inadequate Swedish language skills were the only exclusion criteria. The total participation rate was 40.8%. A detailed description of the cohort has been previously published[11]. The study was approved by the ethical committee at the Lund University (LU 51-90). A written informed consent was provided by all the participants.

For the current study we included participants that were part of the 6,103 randomly selected individuals who underwent additional phenotyping during 1991-1994 as part of the MDCS- Cardiovascular Cohort (MDCS-CC), designed to study epidemiology of carotid artery. In total 3,734 MDCS-CC participants, of those that were alive and had not emigrated from Sweden (n=4924), attended a follow-up re-examination between 2007 and 2012, which has been described previously [12]. Urinary albumin-creatinine concentration was quantified from overnight urine using Cobas at the Department of Clinical Chemistry. We included 2,641 participants, who had complete data on urinary albumin-creatinine concentration at follow-up re-examination and with non-missing genotype data. Of these, in total 547 had a history of either a prevalent or incident diabetes at follow-up re-examination.

SUMMIT Consortia Studies

CAMBRIDGE / ADOLESCENT TYPE 1 DIABETES CARDIO-RENAL INTERVENTION TRIAL (ADDIT) [13]

As part of preliminary screening for a multicentre, randomized controlled trial of statins/ACE inhibitors, we measured albumin-creatinine ratio (ACR) in 6 early morning urine samples from 3,353 adolescents (10-16 years) and calculated tertiles based on an established algorithm. From those subjects deemed to be at higher risk (upper ACR tertile) we recruited 400 into the intervention study (Trial cohort). At baseline vascular measurements (carotid intima-media thickness, pulse wave velocity (PWV), flow-mediated dilatation, digital pulse amplitude tonometry), renal (symmetric dimethylarginine, cystatin C, creatinine) and CVD markers (lipids and apolipoproteins (Apo)A-1 and B, C-Reactive Protein, asymmetric dimethylarginine) were assessed. The trial cohort were genotyped on the Illumina omniexpress SNP genotyping array as part of the SUMMIT study of renal complications in subjects with diabetes[13].

EURODIAB [13]

A total of 3250 men and women with type 1 diabetes were recruited from 31 centers in 16 European countries, and were aged between 15 and 60 at the baseline investigation phase (1989 to 1991). The diagnosis of type 1 diabetes was a clinical one; diagnosis had to have occurred in the patient prior to age 36 and the patient had a continuous need for insulin within a year of diagnosis. Re-examination occurred on average of between six and eight years after baseline investigations. The trial was designed to investigate the causes of complications in subjects with type 1 diabetes. Renal phenotypes were defined using the criteria detailed in van Zuydam et al., 2018[13].

FINNDIANE [14-16]

The Finnish Diabetic Nephropathy (FinnDiane) Study is an ongoing nationwide multicenter study of individuals with type 1 diabetes, with the aim to identify clinical, environmental and genetic risk factors for diabetic complications and diabetic nephropathy in special. The study was established in 1997, and new individuals with type 1 diabetes continue to be added to the study; currently, more than 5000 individuals are included in the study. The study protocol has been previously described[14]. At their baseline visit, all patients went through a comprehensive clinical examination and the patients' attending physician filled the standardized questionnaires regarding health and medical history. Patients gave also urine and blood samples used to measure e.g. albumin excretion rate (AER). A subset of patients came also for one or more follow-up visits with similar setting. Further information on clinical events has been retrieved from the national hospital discharge register (National Care Register of Health Care). In addition, over 2000 Finnish individuals with type 1 diabetes were added to the FinnDiane study through collaboration with the National Institute of Health

and Welfare (Finland). For these, health related information was retrieved from the patients' medical records as well as from the national registries.

The genome-wide genotyping of 3651 FinnDiane individuals was performed as previously described [15, 16]. Following the common analysis plan for the SUMMIT cohorts, AER was measured from 24-hour urine collections, and taken from the latest visit, but excluding visits after ESRD (defined as dialysis or Transplant or eGFR<15). If multiple 24-hour AER measurements were available within one year, the geometric mean of these values was calculated to reduce the effect of day-to-day variability. The AER values were log-transformed. Only patients with a minimum diabetes duration of 10 years were included in the analysis. A total of 2840 individuals fulfilled the inclusion criteria, had required genetic and phenotypic data available, and were included in the analysis. Analysis was adjusted for use of anti-hypertensive medication, sex, age at diabetes onset, diabetes duration, and principal components.

GODARTS [17-19]

The Genetics of Diabetes and Audit Research in Tayside Scotland (GoDARTS 1 and GoDARTS 2) is a case controls study of type 2 diabetes that includes ~10,000 subjects with diabetes and ~6000 subjects with no history of diabetes. A full description of study recruitment is given in Morris et al., 1997[17]. Subjects with T2D included in the study of kidney related phenotypes were either genotyped on the Affymetrix 6.0 SNP array or the Illumina Omniexpress array. All subjects were linked to electronic medical records including hospital admission and blood biochemistry. These data were used to derive renal phenotypes as described in van Zuydam et al. 2018[13].

BENEDICT (PHASE A and B) [20, 21]

The multicenter double-blind, randomized Bergamo Nephrologic Diabetes Complications Trial-A (BENEDICT) was designed to assess whether angiotensin-converting-enzyme inhibitors and non-dihydropyridine calcium-channel blockers, alone or in combination, prevent microalbuminuria in subjects with hypertension, type 2 diabetes mellitus, and normal urinary albumin excretion. We studied 1204 subjects, who were randomly assigned to receive at least three years of treatment with trandolapril (at a dose of 2 mg per day) plus verapamil (sustained-release formulation, 180 mg per day), trandolapril alone (2 mg per day), verapamil alone (sustained-release formulation, 240 mg per day), or placebo. The target blood pressure was 120/80 mm Hg. The primary end point was the development of persistent microalbuminuria (overnight albumin excretion, > or =20 microg per minute at two consecutive visits) [20].

The Bergamo Nephrologic Diabetes Complications Trial-B was a multicentre, prospective, double-blind, parallel-group trial comparing renal and cardiovascular outcomes in 281 hypertensive type 2 diabetes patients with microalbuminuria randomized to at least 2-year VeraTran (verapamil/trandolapril 180 mg/2 mg daily) or trandolapril (2 mg daily, identical image) treatment. Main outcome was persistent macroalbuminuria (albuminuria >200 µg/min in two consecutive visits). Treatment targets were SBP/DBP less than 120/80 mmHg and HbA1C less than 7%[21].

SCANNIA DIABETES REGISTRY (SDR) [16, 19]

Patients in SDR were randomly collected from the Department of Endocrinology, Malmö Sweden and surrounding clinics in Skåne (Scania) Sweden 3. The total cohort included 7414 individuals with all types of diabetes. Diabetes classification into type 1 diabetes and type 2 diabetes was done based on presence of GAD antibodies and c-peptide levels, or in case of incomplete information, based on the diagnosis given by the treating physician. Patients were selected for genotyping based on presence of complications (kidney disease

or retinopathy) or absence of complications in spite of more than 15/10 years duration of diabetes for T1D/T2D respectively [16]. Patients of known non-Scandinavian origin were excluded from the analysis.

STENO TYPE 2 DIABETES [13, 19]

These comprise a sample of clinical-onset type 2 diabetes patients and non-diabetic control individuals who were examined at the outpatient clinic at Steno Diabetes Center, Copenhagen. An OGTT in all control individuals confirmed the exclusion of individuals with unknown diabetes or states of pre-diabetes according to WHO 1999 criteria.

Replication in Greenlanders

GREENLANDERS [22]

The Greenlandic samples are from two different cohorts: B99 (N = 1,401) and Inuit Health in Transition (IHIT) (N = 3,115), which were collected in Greenland as a part of general population health surveys conducted in 1999–2001 and 2005–2010, respectively.

The current study comprises a total of 2,605 individuals (IHIT=2,519; B99=86) with complete genotype and albuminuria measurements available.

ESM Methods 1.3 Danish Exome Sequencing based SNP Selection

16, 340 coding SNPs selected from the Danish Exome sequencing study comprising 1000 T2D patients and 1000 matched controls were genotyped as part of the custom designed Illumina iSelect array [23, 24]. SNPs based on the following 3 criterion were selected from the exome sequencing study: 1) annotated to the most likely deletiriou cateogries (nonsense, nonsynonymous, located in splice sites, and in untranslated regions, n=14,654); 2) nominally associated with T2D in the exome sequencing based association (n=995); 3) additional coding variants and lead SNP in 192 loci associated with common metabolic traits at genome wide significance (n=700) [23]. Genotype calling was performed using Gentrain 1.0 clustering algorithm based on a custom cluster file created using 1032 samples with high quality data selected from various batches as described [23]. SNP quality filters included minor allele frequency (MAF>0.01), genotype call rate >95% and Hardy –Weinberg equilibrium ($P > 10^{-7}$). Individuals who were related with inbreeding coefficients (>0.1 or < -0.1), or a low call rate (<95%), or mislabeled sex were removed. More details have been reported previously [23].

ESM Methods 1.4 Analyses Plan: Albuminuria Exome Chip based Exome Wide Association Study

Introduction

AIM: This analysis plan aims to coordinate collection of summary statistics data of Exome Chip based analyses (Exome wide association study) for the trait "**Urinary Albumin Creatinine Ratio/ACR**" or "**Urinary albumin excretion rate/AER**" (mg/g or mg/24 hours) as a continuous variable.

We aim to carry out replication analyses of the provided list of SNPs (Exome Chip based)

Please contact **Tarunveer S. Ahluwalia (veertarun@gmail.com or Tarun.veer.singh.ahluwalia@regionh.dk)** if you have any questions regarding trait definitions or analyses implementation.

General guidelines for analyses

Each individual study will perform data quality control (QC) and analysis and provide summary results for meta-analysis.

Outcome

- **ACR**: albumin creatinine ratio (mg/g) or **AER:** Albumin excretion rate (mg/24 hrs.)

Exclusions

- All individuals less than 18 years of age

Stratified analyses

- Ethnicity: Perform all analyses stratified by self-reported race/ethnicity (Caucasians/Europeans, African Americans, etc.)
- Diabetes (DM) status: Perform analyses stratifying for diabetes status, as well as combined analyses pooling all data.

Pre Analyses Sample Quality Control (QC) including but not limited to:

- Exclude individuals with more than > 5% missing markers
- Exclude ethnicity outliers based on population clustering
- Exclude gender mismatches
- For duplicate and twin pairs, keep the individual with less missing data

NOTES:

- For twin studies/family data, we recommend that data from one individual from each twin pair/family (preferably with a higher genotyping call rate and preferably if the sample is a case) be used.

- We will refer to principal components (PCs), which are used to control for population stratification and other confounders. Here, we don't distinguish between PCs generated using software like smartPCA/EIGENSOFT or components calculated using multidimensional scaling (MDS) as implemented in PLINK. For your analyses, you should include either PCs or MDS components as covariates in the models. **Principal components in studies with related individuals to be calculated in founders.**

- Limit your dataset to individuals with valid data for phenotype, genotype, covariates, and PCs.

Analyses

Trait Transformation

Take the the natural logarithm of ACR/AER use this as the outcome variable (*ln_acr or ln_aer*)

**Models to be run, single-marker and gene-based:*

1. **All individuals**
   Outcome (ln_aer/acr) ~ age + sex + SNP + PCs (if appropriate) + study specific covariates (if appropriate)
2. **Individuals with Diabetes**
   Outcome (ln_aer/acr) ~ age + sex + SNP + PCs (if appropriate) + study specific covariates (if appropriate)
3. **Individuals without diabetes**
   Outcome (ln_aer/acr) ~ age + sex + SNP + PCs (if appropriate) + study specific covariates (if appropriate)

*Adjust for Systolic Blood Pressure and DM duration wherever available/relevant.

Gene-based Analyses

- Each study needs to provide meta SSD (.MSSD) and meta Info (.MInfo) files generated from the genotype (binary plink) and .setID file (which has Gene set name and SNP name) using the METASkat Package. This will later be used to run the meta analyses by us. The setID file will be provided by us
- Cohorts should not use a frequency threshold when running SKAT tests. Filters will be applied at the meta-analysis stage
- For each trait, please generate (.MSSD) and (.MInfo) files in case stratified manner and for the pooled analyses

NOTE: There has been some SNP filtering based on functionality performed at our center and therefore we will provide you with the same list of markers to be used for this analyses (which can be used to create a new binary file before proceeding to step 1 of METAskat)

Example R code:

```
library("MetaSKAT")
library("SKAT")

family_file_with_phenotype    <- read.table("cohort.fam")
n_sample                                              <- nrow(family_file_with_phenotype)

cohort_null                                    <- SKAT_Null_Model(family_file_with_phenotype$ln_uaer~1)

Generate_Meta_Files(cohort_null, "cohort.bed", "cohort.bim", "SKAT_sets.txt", "cohort.MSSD",
"cohort.MInfo", n_sample)
```

Summary Data for single-marker analyses

An Excel Sheet asking for summary data will be provided to all participating cohorts where information on data summary, genotyping summary, author list, a brief study description and acknowledgements for each study would be required. This file can then be completed by each study group and uploaded on the server details provided for data upload as “Exome.descriptive.STUDYNAME.xls”.

Data upload format for single SNP analyses with BUILD GRCh 37, Hg19:

**CHR**	**Chromosome**
**MARKER ID**	**Chip marker ID (exm…., rs….)**
**rsID**	**rs number if available**
**POS**	**Chromosomal position of SNP in BP**
**EFFECT_ALLELE**	**Allele associated with effect**
**NON_EFFECT_ALLELE**	**Non-effect allele**
**EAF**	**Effect allele frequency**
**N**	**Total number of individuals tested**
**BETA**	**Beta value**
**SE**	**Standard Error**
**PVAL**	**P-value**
**IMPUTATION**	**0=genotyped; 1=imputed**
**IMPUTATION_QUALITY**	**Eg. r2 for MACH (Numeric)**

File naming convention

Please use the following naming scheme:
STUDY.ANALYSES.ETHNICITY.DATE.ANALYST_INITIALS.txt

1.

STUDY is a short (14 characters or less) identifier for the population studied. ANALYSES pertains to models: “POOLED”, “DM”, or ”NDM”. ETHNICITY: European American (EA), European (EA), African American (AA), Greenlandic (GL). DATE in format ddmmyy. INITIALS (analysts).
Eg STENO.POOLED.EA.240615.TSA.txt
STENO.DM.EA.240615.TSA. txt

References to ESM Methods

1. Rasmussen SS, Johansen NB, Witte DR, *et al.* Incidence of register-based diabetes 10 years after a stepwise diabetes screening programme: the ADDITION-Denmark study. Diabetologia 2016;59(5):989-997
2. Jensen TM, Vistisen D, Fleming T, *et al.* Methylglyoxal is associated with changes in kidney function among individuals with screen-detected Type 2 diabetes mellitus. Diabet Med 2016;33(12):1625-1631
3. Thuesen BH, Cerqueira C, Aadahl M, *et al.* Cohort Profile: the Health2006 cohort, research centre for prevention and health. Int J Epidemiol 2014;43(2):568-575
4. Aadahl M, Zacho M, Linneberg A, *et al.* Comparison of the Danish step test and the watt-max test for estimation of maximal oxygen uptake: the Health2008 study. Eur J Prev Cardiol 2013;20(6):1088-1094
5. Jorgensen T, Borch-Johnsen K, Thomsen TF, *et al.* A randomized non-pharmacological intervention study for prevention of ischaemic heart disease: baseline results Inter99. Eur J Cardiovasc Prev Rehabil 2003;10(5):377-386
6. Skaaby T, Husemoen LL, Ahluwalia TS, *et al.* Cause-specific mortality according to urine albumin creatinine ratio in the general population. PLoS One 2014;9(3):e93212
7. Petersen ER, Nielsen AA, Christensen H, *et al.* Vejle Diabetes Biobank - a resource for studies of the etiologies of diabetes and its comorbidities. Clin Epidemiol 2016;8:393-413
8. Dantoft TM, Ebstrup JF, Linneberg A, *et al.* Cohort description: The Danish study of Functional Disorders. Clin Epidemiol 2017;9:127-139
9. Hadjadj S, Cariou B, Fumeron F, *et al.* Death, end-stage renal disease and renal function decline in patients with diabetic nephropathy in French cohorts of type 1 and type 2 diabetes. Diabetologia 2016;59(1):208-216
10. Charmet R, Duffy S, Keshavarzi S, *et al.* Novel risk genes identified in a genome-wide association study for coronary artery disease in patients with type 1 diabetes. Cardiovasc Diabetol 2018;17(1):61
11. Berglund G, Elmstahl S, Janzon L, *et al.* The Malmo Diet and Cancer Study. Design and feasibility. J Intern Med 1993;233(1):45-51
12. Rosvall M, Persson M, Ostling G, *et al.* Risk factors for the progression of carotid intima-media thickness over a 16-year follow-up period: the Malmo Diet and Cancer Study. Atherosclerosis 2015;239(2):615-621
13. van Zuydam NR, Ahlqvist E, Sandholm N, *et al.* A Genome-Wide Association Study of Diabetic Kidney Disease in Subjects With Type 2 Diabetes. Diabetes 2018
14. Thorn LM, Forsblom C, Fagerudd J, *et al.* Metabolic syndrome in type 1 diabetes: association with diabetic nephropathy and glycemic control (the FinnDiane study). Diabetes Care 2005;28(8):2019-2024
15. Sandholm N, Salem RM, McKnight AJ, *et al.* New susceptibility loci associated with kidney disease in type 1 diabetes. PLoS Genet 2012;8(9):e1002921
16. Sandholm N, Van Zuydam N, Ahlqvist E, *et al.* The Genetic Landscape of Renal Complications in Type 1 Diabetes. J Am Soc Nephrol 2017;28(2):557-574
17. Morris AD, Boyle DI, MacAlpine R, *et al.* The diabetes audit and research in Tayside Scotland (DARTS) study: electronic record linkage to create a diabetes register. DARTS/MEMO Collaboration. BMJ 1997;315(7107):524-528
18. Hebert HL, Shepherd B, Milburn K, *et al.* Cohort Profile: Genetics of Diabetes Audit and Research in Tayside Scotland (GoDARTS). Int J Epidemiol 2018;47(2):380-381j
19. Alkayyali S, Lajer M, Deshmukh H, *et al.* Common variant in the HMGA2 gene increases susceptibility to nephropathy in patients with type 2 diabetes. Diabetologia 2013;56(2):323-329
20. Ruggenenti P, Fassi A, Ilieva AP, *et al.* Preventing microalbuminuria in type 2 diabetes. N Engl J Med 2004;351(19):1941-1951

21. Ruggenenti P, Fassi A, Ilieva A, *et al.* Effects of verapamil added-on trandolapril therapy in hypertensive type 2 diabetes patients with microalbuminuria: the BENEDICT-B randomized trial. J Hypertens 2011;29(2):207-216
22. Grarup N, Moltke I, Andersen MK, *et al.* Loss-of-function variants in ADCY3 increase risk of obesity and type 2 diabetes. Nat Genet 2018;50(2):172-174
23. Albrechtsen A, Grarup N, Li Y, *et al.* Exome sequencing-driven discovery of coding polymorphisms associated with common metabolic phenotypes. Diabetologia 2013;56(2):298-310
24. Ahluwalia TS, Allin KH, Sandholt CH, *et al.* Discovery of coding genetic variants influencing diabetes-related serum biomarkers and their impact on risk of type 2 diabetes. J Clin Endocrinol Metab 2015;100(4):E664-671
25. Stevens LA, Coresh J, Greene T, *et al.* Assessing kidney function--measured and estimated glomerular filtration rate. N Engl J Med 2006;354(23):2473-2483
26. Mahajan A, Wessel J, Willems SM, *et al.* Refining the accuracy of validated target identification through coding variant fine-mapping in type 2 diabetes. Nat Genet 2018;50(4):559-571

II. Electronic Supplementary Material (ESM) Tables

Index

**ESM Table #**	**Contents**
**1.**	Study Albuminuria measurement Methods
**2.**	Study Genotyping Details
**3.**	Study characteristics stratified by index (discovery) SNP genotypes
**4.**	SNP-albuminuria associations adjusted for Systolic Blood Pressure
**5.**	Conditional analyses of the *CUBN* rs141640975
**6.**	Detailed results for the genes identified in the gene aggregate tests
**7.**	Association of *CUBN* rs141640975 with type 2 diabetes and kidney function (eGFR)
**8.**	Known GWAS SNP-trait associations for *KCNK5* rs10947789
**9.**	Known eQTL based associations for *KCNK5* rs10947789
**10.**	Known metabolite-SNP associations for *KCNK5* rs10947789 & *CUBN* rs141640975

ESM table 1. Albuminuria measurement methods

**STUDY NAME**	**Method for measurement of Albuminuria (Albumin excretion rate (AER) or Albumin creatinine ratio (ACR))**	**Cohort specific AER deifinition**
**Discovery**		
Inter99	Urine albumin concentration was analysed by a turbidimetric assay (Cobas Mira plus, Roche diagnostic systems, la Roche, Basel, Switzerland). Urine creatinine concentration was analysed by the Jaffé method (Hitachi 912 system, Roche diagnostic, Germany).	-
Health2006	Urinary albumin concentration was measured using turbidimetry (Hitachi 912, Roche Diagnostics, Germany) with reagents from DAKO (Glostrup, Denmark). Urinary creatinine concentration was measured by the Jaffé method (Hitachi 912, Roche Diagnostics, Germany).	-
Health2008	Urinary albumin concentration was measured using turbidimetry (Hitachi 912, Roche Diagnostics, Germany) with reagents from DAKO (Glostrup, Denmark). Urinary creatinine concentration was measured by the Jaffé method (Hitachi 912, Roche Diagnostics, Germany).	-
Vejle biobank	Urinary albumin concentration (mg/L) was measured using turbidimetry (Cobas 8000, module cobas c502, Roche Diagnostics, Germany) with reagents from DAKO (Glostrup, Denmark). Urinary creatinine concentration was measured by the enzymatic method (Cobas 8000, module cobas c702, Roche Diagnostics, Germany).	-
ADDITION-DK	Urinary albumin and Creatinine levels were quantified using, Quantitative immunologic analysis by turbidimetry on Hitachi 912 system,Roche Diagnostics, Germany.	-
**REPLICATION**	**Method for measurement of Albuminuria (Albumin excretion rate (AER) or Albumin creatinine ratio (ACR))**	
MDC	Urinary albumin-creatinine concentration was quntified from overnight urine sample using (Cobas Mira plus, Rosche diagnostic systems, la Roche, Basel, Switzerland), at the Department of Clinical Chemistry, Malmo, Sweden	-
**Genesis/GENDIAB**	Urinary albumin : immunonephelometry; urine creatinine: modified Jaffé method	-
**DanFunD**	Urinary albumin was measured by turbidometry using the Vitros 5600 (Ortho Clinical Diagnostics, Illkirch Cedex, France). Urinary creatinine was measured by Vitros 5600 (Ortho Clinical Diagnostics, Illkirch Cedex, France).	-
**Greenlandic Inuit**	Urinary albumin and Creatinine levels were quantified using, Quantitative immunologic analysis by turbidimetry on Hitachi 912 system,Roche Diagnostics, Germany.	-
**SUMMIT Cohorts**		AER (Definition)

<table>
<tr><td>Eurodiab</td><td>Aliquots were frozen and sent to London for analysis of urinary albumin, using an immunoturbidimetric method10 that included goat anti-human albumin antisera (Sanofi Diagnostics Pasteur Inc., MN, USA) and human serum albumin standards (ORHA 20/21 grade HSA; Behring Diagnostics, Hoechst UK Ltd., Hounslow, Middlesex, UK).</td><td rowspan="8">ln(AER) or ln(ACR), not raw values.
Exclude values during ESRD (defined as dialysis or Transplant or eGFR<15)
Reason: AER starts to decrease before the onset of dialysis treatment, when eGFR is very low and the kidneys do not filter any more. Time point: Latest visit before ESRD
If this is not feasible, then the baseline value, excluding patients with ESRD. Preference on 24-h AER, or the measurement with the most data (24-h AER, overnight AER, or ACR) if significantly more than 24h AER
If possible, the two different types of collections (24-h and overnight AER) should not be mixed together within one cohort (or within one patient, if multiple measurements are available).
If large amounts of both types of AER exist, then these patients can be analyzed separately. No patient should be analyzed twice, even if multiple types of AER/ACR exist.
Min duration 10 years (T1D) or 5 years (T2D)
Reason: Long enough duration ensures that the patients had enough time to develop symptoms of nephropathy
Adjusted for AHT medication if available
T2D: Nearly all the patients have AHT medication. Thus, adjusting for it may be unnecessary.
Other covariates: Sex, age at diabetes onset, diabetes duration
Mean of all measurements within 1 year of the visit (if available)
Reduces the effect of day-to-day variability
Use the mean of ln(AER): [ln(AER1) + ln(AER2) + ln(AER3) + …]/N_AER.
Do not use ln of mean AER: ln([AER1 + AER2 + AER3 + …]/N_AER).</td></tr>
<tr><td>FinnDiane</td><td>Albuminuria was measured from a 24-h urine collection at the local healt care centre, or at the FinnDiane visit. Geometric mean of albuminuria was used if multiple (up to 4) measures were available within one year. Serum creatinin was measured using a modified Jaffe method for samples before 2002, and enzymatic colorimetric method thereafter. Values measured with Jaffe method were corrected to correspond the enzymathic method.</td></tr>
<tr><td>SDR</td><td>Urinary albumin was measured at the clinical chemistry department as part of the patients routine care using turbidimetry on Cobas (Roche). Serum creatinine was measured using an enzymatic colorimetric method on Cobas (Roche)</td></tr>
<tr><td>Steno type 2 diabetes</td><td>Albuminuria was measured from a 24-h urine collection at Steno Diabetes Centre Copenhagen.</td></tr>
<tr><td>GoDARTS 1</td><td>Albumin excretion rate measures were all performed in the clinical biochemistry laboratory at Ninewells Hospital, Dundee</td></tr>
<tr><td>GoDARTS2</td><td>Albumin excretion rate measures were all performed in the clinical biochemistry laboratory at Ninewells Hospital, Dundee</td></tr>
<tr><td>Cambridge</td><td>Urinary albumin and creatinine were measured using the turbidometric/Jaffe Method.</td></tr>
<tr><td>BENEDICT STUDY PHASE A AND B</td><td>Albuminuria was measured from overnight urine collection.</td></tr>
</table>

		Values below detection limit, or the 0 values<br>ln(0) = -Inf. Thus, we have to convert the zero values to non-zero values.<br>Suggestion: use a fixed value that is slightly smaller than the smallest value otherwise: 24h-AER: 0.1 mg/24h; nU-AER: 0.01 µg/min; ACR: 0.01 mg/mmol<br>Or use the smallest value available in the data set<br>or use the detection limit

ESM table 2. Genotyping and QC Summary: Discovery and Replication Cohorts.

Study_type	**Diabetes Type**	**Cohort**	**Full study name**	**Genotyping centre**	**Genotyping array**	**Calling algorithm**	**Pre-Imputation QC - Exclusion criteria**	**Sample call rate**	**SNP call rate**
Discovery									
Population based	2	Inter99	Inter99	NNF CBMR, Copenhagen	Illumina Human Exome v12_1.0	GenomeStudio & Z Call	SNP call rate <98%, HWE p<10-6, cluster separation score <0.4. Sample call rate <95%, gender check, genetic duplicates and non European origin (Non CEU) removed	95%	98%
Population based	2	Health2006	Health2006	NNF CBMR, Copenhagen	Illumina Human Exome v12_1.0	GenomeStudio & Z Call	SNP call rate <98%, HWE p<10-6, cluster separation score <0.4. Sample call rate <95%, gender check, genetic duplicates and non European origin (Non CEU) removed	95%	98%
Population based	2	Health2008	Health2008	NNF CBMR, Copenhagen	Illumina Human Exome v12_1.0	GenomeStudio & Z Call	SNP call rate <98%, HWE p<10-6, cluster separation score <0.4. Sample call rate <95%, gender check, genetic duplicates and non European origin (Non CEU) removed	95%	98%
Diabetes Cases	2	ADDITION-DK	Anglo–Danish–Dutch Study of Intensive Treatment in People with Screen-Detected Diabetes in Primary Care	NNF CBMR, Copenhagen	Illumina Human Exome v12_1.0	GenomeStudio & Z Call	SNP call rate <98%, HWE p<10-6, cluster separation score <0.4. Sample call rate <95%, gender check, genetic duplicates and non European origin (Non CEU) removed	95%	98%
Diabetes Cases	2	Vejle biobank	-	NNF CBMR, Copenhagen	Illumina Human Exome v12_1.0	GenomeStudio & Z Call	SNP call rate <98%, HWE p<10-6, cluster separation score <0.4. Sample call rate <95%, gender check, genetic duplicates and non European origin (Non CEU) removed	95%	98%
Replication									
Population based	2	MDCS	Malmö Diet and Cancer Study	Broad Institute, Cambridge, MA USA.	Illumina HumanOmniExpress Exome BeadChip v. 1.0	GenomeStudio v2011.1	1. bad call rate<br>2. excess homozygosity<br>3. failed gender check<br>4. Related individuals/duplicates<br>5. Popoulation outliers<br>6. monormorphic SNPs	95%	95%
Diabetes Cases	1	Genesis/Genediab			Illumina Human Exome v12_1.0	Genome studio	bad call rate, failed gender check, related individuals/duplicates, population outliers, monomorphic SNPs	95%	99%
Population based	2	DanFunD	The Danish study of Functional Disorders	DeCode Genetics, Reykjavik, Iceland	Illumina Omni Express	Genome studio Gencall	SNP call rate 98%, HWE p 10-5	95%	98%

Population based		Greenlander Cohorts (IHIT, B99)			Illumina Metabochip	GenomeStudio	Duplicates removed, sample call rate <98%, Gender discrepanecy, SNPs_MAF<1%, HWE p<10e-10, SNPs associated with gender p<10e-05	98%	98%
IMI-SUMMIT Consortia Replication cohorts									
Diabetes cases	1	Eurodiab	EURODIAB Family Study (a substudy of the EURODIAB PCS)	Lund University	Illumina Omni-express	GenomeStudio	SNP call rate <95%, MAF<1%, HWE p<10-6, evidence of plate differences (p<1e-7). Sample call rate <95%, extremely high/low heterozygosity (>3 sd. from mean), Admixture (PC1 or PC2 > 6 sd. away from mean + visual evaluation),	99%	99%
Diabetes cases	1	FinnDiane	Finnish Diabetic Nephropathy Study	FIMM	Human 610Quad	GenomeStudio	SNP call rate <95%, MAF<1%, HWE p<10-7, missingness by haplotype (p<10-8), missingness by phenotype (p<1e-8), evidence of plate differences (p<1e-7). Sample call rate <95%, extremely high/low heterozygosity (>4 sd. from mean), first degree relatives, Admixture (PC1 or PC2 > 6 sd. away from mean + visual evaluation),	99.80%	99.90%
Diabetes cases	1	Cambridge		Lund University	Illumina Omni-express	GenomeStudio	SNP call rate <95%, MAF<1%, HWE p<10-6, evidence of plate differences (p<1e-7). Sample call rate <95%, extremely high/low heterozygosity (>3 sd. from mean), Admixture (PC1 or PC2 > 6 sd. away from mean + visual evaluation),	99%	99%
Diabetes cases	1	SDR1	Scannia Diabetes Registry	Lund University	Illumina Omni-express	GenomeStudio	SNP call rate <95%, MAF<1%, HWE p<10-6, evidence of plate differences (p<1e-7). Sample call rate <95%, extremely high/low heterozygosity (>3 sd. from mean), Admixture (PC1 or PC2 > 6 sd. away from mean + visual evaluation),	99%	99%
Diabetes cases	2	SDR2	Scannia Diabetes Registry	Lund University	Illumina Omni-express	GenomeStudio	SNP call rate <95%, MAF<1%, HWE p<10-6, evidence of plate differences (p<1e-7). Sample call rate <95%, extremely high/low heterozygosity (>3 sd. from mean), Admixture (PC1 or PC2 > 6 sd. away from mean + visual evaluation),	99%	99%
Diabetes cases	2	BENEDICT STUDY PHASE A AND B	BENEDICT, DEMAND, Mario Negri	Lund University	Illumina Omni-express	GenomeStudio	SNP call rate <95%, MAF<1%, HWE p<10-6, evidence of plate differences (p<1e-7). Sample call rate <95%, extremely high/low heterozygosity (>3 sd. from mean), Admixture (PC1 or PC2 > 6 sd. away from mean + visual evaluation),	99%	99%
Diabetes cases	2	STENO type 2 diabetes		Lund University	Illumina Omni-express	GenomeStudio	SNP call rate <95%, MAF<1%, HWE p<10-6, evidence of plate differences	99%	99%

							(p<1e-7). Sample call rate <95%, extremely high/low heterozygosity (>3 sd. from mean), Admixture (PC1 or PC2 > 6 sd. away from mean + visual evaluation),		
Diabetes cases	2	GoDARTS 1	Genetics of Diabetes Audit Research Tayside Scotland	Oxford University	Affymetrix 6.0 SNP genotyping array	CHIAMO	SNP call rate <95%, MAF<1%, HWE p<10-6, evidence of plate differences (p<1e-7). Sample call rate <95%, extremely high/low heterozygosity (>3 sd. from mean), Admixture (PC1 or PC2 > 6 sd. away from mean + visual evaluation),	99%	99%
Diabetes cases	2	GoDARTS2	Genetics of Diabetes Audit Research Tayside Scotland	Lund University	Illumina Omniexpress	GenomeStudio	SNP call rate <95%, MAF<1%, HWE p<10-6, evidence of plate differences (p<1e-7). Sample call rate <95%, extremely high/low heterozygosity (>3 sd. from mean), Admixture (PC1 or PC2 > 6 sd. away from mean + visual evaluation),	99%	99%

ESM table 3. Study characteristics stratified by lead (discovery) SNP genotypes

**Characteristics**	***CUBN* rs141640975**		
	**GG**	**GA**	**AA**
N	13,076	218	2
*Age (years)	51.5 ± 11.3	50.9 ± 11.6	53.0 ± 16.8
Women (%)	49.5	45.4	0
*BMI (Kg/m²)	27.6 ± 5.4	28.1 ± 5.8	27.3 ± 8.2
*SBP (mmHg)	134.8 ± 19.6	134.6 ± 19.8	155.5 ± 7.7
*DBP (mmHg)	82.9 ± 10.9	82.3 ± 11.2	90.2 ± 6.7
**UACR (mg/mmol)	0.45 (0.33-0.90)	0.56 (0.33-1.12)	20.01 (13.6-26.4)
*S. Creat. (mg/dl)	0.87 ± 0.21	0.86 ± 0.17	-
Smokers (%)	62.4	55.7	0
	***KCNK5* rs10947789**		
	**TT**	**TC**	**CC**
N	7,801	4,714	781
*Age (years)	51.4 ± 11.4	51.6 ± 11.3	50.9 ± 11.2
Women (%)	49.0	49.7	52.4
*BMI (Kg/m²)	27.6 ± 5.4	27.7 ± 5.5	27.6 ± 5.5
*SBP (mmHg)	134.9 ± 19.8	134.7 ± 19.6	133.4 ± 18.5
*DBP (mmHg)	83.1 ± 11.0	82.8 ± 10.9	82.1 ± 10.6
**UACR (mg/mmol)	0.45 (0.33-0.84)	0.45 (0.33-0.90)	0.45 (0.33-0.99)
*S. Creat. (mg/dl)	0.87 ± 0.19	0.86 ± 0.23	0.86 ± 0.18
Smokers (%)	62.2	62.2	64.1
	***LMX1B* rs140177498**		
	**CC**	**CT**	**TT**
N	13,068	228	0
*Age (years)	51.4 ± 11.3	51.6 ± 10.9	-
Women (%)	49.5	49.1	-
*BMI (Kg/m²)	27.7 ± 5.4	27.4 ± 5.6	-
*SBP (mmHg)	134.8 ± 19.6	135.4 ± 21.5	-
*DBP (mmHg)	82.9 ± 10.9	83.3 ± 11.4	-
**UACR (mg/mmol)	0.45 (0.33-0.90)	0.56 (0.33-1.16)	-
*S. Creat. (mg/dl)	0.87 ± 0.21	0.88 ± 0.25	-
Smokers (%)	60.3	61.6	-

*****Data is mean ± standard deviation, ** data is median (inter quartile range); Smokers: % of smokers who have ever smoked or are current smokers (based on smoking data on a subset, n_smoking: 9,294); S.Creat: Serum Creatinine measures based on a total of n: 11,289 individuals at baseline. SBP: Systolic blood pressure based on n=13, 266 individuals, DBP: Diastolic blood pressure based on 13,264 individuals; BMI: basic metabolic index calculated as weight in Kg/height in m², BMI based on a total of n=13,276 individuals. Age and UACR:Urinary albumin creatinine ratio, based on n=13,296 individuals.

ESM table 4. SNP-albuminuria associations adjusted for Systolic Blood Pressure.

Gene	SNP	EA/OA	BETA	SE	P value
CUBN	rs141640975	A/G	0.33	0.065	5.88×10^{-7}
KCNK5	rs10947789	C/T	0.058	0.014	3.11×10^{-5}
LMX1B	rs140177498	T/C	0.26	0.065	6.29×10^{-5}

All SNP trait associations are based on linear regression additive models on pooled genotype data from the discovery set cohorts (n:13,226) and correcting for age, sex, population substructure (PCs1-10) + study cohort + systolic blood pressure (SBP). EAF: Effect allele frequency; SE: Standard Error

ESM table 5. Conditional analyses of the *CUBN* rare SNP

SNP #	SNP	EA/OA	EAF (%)	BETA	SE	P_association	P_condition
1	rs141640975 (A1690V)	A/G	0.83	0.33	0.066	6.01×10^{-7}	
2	rs1801239 (LD with rs17343073,r^2=0.92)	G/A	10	0.068	0.019	5.83×10^{-4}	
3	rs6602163/rs10795433 (proxy: rs62619939, r^2=0.60)	G/C	12	0.062	0.018	8.85×10^{-4}	
	rs141640975_condition_snp #2		0.83	0.34	0.066		2.91×10^{-7}
	rs141640975_condition_snp #3		0.83	0.34	0.066		2.85×10^{-7}

All SNP trait associations are based on linear regression additive models on pooled genotype data from the discovery set cohorts (n=13,226) and correcting for age, sex, population substructure (PCs1-10) + study cohort. EAF: Effect allele frequency. LD r2 between rs1801239 and rs17343073: 0.92, between rs6602136 and rs10795433 is 1.0, between SNP#3 and available proxy rs62619939 is 0.6

ESM table 6. Detailed results for the genes identified in the gene aggregate tests

**Gene Set ID**	**rsID**	**Score**	**Amino Acid change**	**Annotation (GVS)**	**MAF (%)**	**Major/Minor Allele**	**Chr:Pos**
HES1	-	5.4	151/281	Coding synonymous	0.067	G/A	3:193855650
	rs141717320	14.2	GLY, SER 227/281	Missense	0.058	G/A	3:193855858
CDC73	rs148612206	13.4	344/532	Coding synonymous near Splice	0.033	T/G	1:193181196
	rs10921318	63.7	-	Intron	32	A/G	1:193104827
GRM5	rs905646	56.7	-	Intron	12	A/G	11:88353802
	rs142032384	5.2	LEU,PRO 1168/1181	Missense	0.016	A/G	11:88241800
	rs144685614	0.28	VAL,MET 842/1181	Missense	0.016	C/T	11:88300327
	rs117017974	2.4	VAL,ILE 709/1181	Missense	0.016	C/T	11:88300726
	rs61741175	-1.4	THR,MET 453/1181	Missense	8.3e-03	G/A	11:88337922
	rs10831496	-50.9	-	Intron	31	A/G	11:88557991
	rs16914280	-1.7	-	Intron	0.033	C/T	11:88321724

MAF: Minor allele frequency in %; Chr:Pos: Chromosome: Position in Base Pairs (Hg19).

ESM table 7. Association of *CUBN* rs141640975 with type 2 diabetes and kidney function (eGFR)

Gene rsID	Pos (hg19)	Trait	EA/OA	EAF (%)	Beta	SE	P	N ($N_{cases}/N_{controls}$)	Source/PMID	Ancestry
**A. Current study**										
CUBN rs141640975	chr10:16992011	*#Type 2 Diabetes	A/G	0. 8	0.19	0.18	0.30	13,296 (3,873/9,423)	Current study	European (Danish)
		#log(eGFR)	A/G	0. 8	0.026	0.013	0.04	13,280	Current Study	European (Danish)
**B. @DIAGRAM consortium**										
CUBN rs141640975	chr10:16992011	*Type 2 Diabetes	A/G	0. 2	0.14	0.11	0.24	79,838	29632382	European
		**Type 2 Diabetes	A/G	0. 2	0.12	0.11	0.29	81,648	29632382	European

*BMI unadjusted model, **BMI adjusted model. eGFR: estimated glomerular filtration rate (eGFR) calculated using the MDRD equation (eGFR=186 × (serum creatinine) $^{-1.154}$ × (age) $^{-0.203}$ × (0.742 if female)) [25]. eGFR units in ml/min./1.73m2 have been natural log transformed. #Logistic/linear model adjusted for age, sex and study center. @[26], EA/OA: Effect allele/Other allele, EAF: Effect allele frequency in %, SE: Standard error, P: p value for association.

ESM table 8. Known GWAS SNP-trait associations for *KCNK5* rs10947789

rsID	Pos (hg19)	Trait	PMID	Year of Publication	Ancestry	Source	EA/OA	EAF (%)	Beta	SE	P	P Het	N
rs10947789	chr6:39174922	Coronary artery disease	28530674	2017	Mixed	Howson JMM	C/T	23	-0.0526	0.0087	1.47E-09	NA	244558
		Coronary artery disease	28530674	2017	European	Howson JMM	C/T	24	-0.0551	0.0091	1.61E-09	NA	218715
		Myocardial infarction	26343387	2015	Mixed	CARDIoGRAMplusC4D	C/T	22	-0.05923	0.01229	1.45E-06	0.30776	167181
		log(Urinary albumin creatinine ratio)	26631737	2016	European	CKD-Gen	C/T	22	0.025	0.0074	0.00056	NA	54449
		log(Urinary albumin creatinine ratio in non diabetics)	26631737	2016	European	CKD-Gen	C/T	22	0.022	0.0076	0.0031	NA	46061
		Visual refractive error	23474815	2013	European	Stambolian D	C/T	NA	0.1114	0.0377	0.00316	NA	7280

EA: Effect allele, OA: Other allele; EAF: Effect allele frequency, P: P value, SE: Standard error

ESM table 9. Known eQTL based associations for *KCNK5* rs10947789

rsID	Pos (hg19)	Data Base (source)	PMID	Ancestry	Tissue	Ensembl	N	EA/OA	EAF (%)	Beta	SE	P
rs10947789	chr6:39174922	GTEx	25954001	Unspecified	Adrenal gland	ENSG00000164626.8	126	C/T	NA	0.5151	0.1167	2.50E-05
	chr6:39174922	GTEx	25954001	Unspecified	Adipose subcutaneous	ENSG00000164626.8	298	C/T	NA	0.3454	0.08564	7.28E-05
	chr6:39174922	MuTHER	22941192	European	Lymphoblastoid cell lines	NA	777	C/T	23	0.03721	0.01195	0.0019
	chr6:39174922	GTEx	25954001	Unspecified	Nerve tibial	ENSG00000164626.8	256	C/T	NA	0.1522	0.05011	0.0026

EA: Effect allele, OA: Other allele; EAF: Effect allele frequency, P: P value, SE: Standard error

ESM table 10. Known metabolite SNP associations for *KCNK5* rs10947789 & *CUBN* rs141640975

Gene	rsID	Pos (hg19)	Trait	PMID	Ancestry	EA/OA	EAF (%)	Beta	SE	P	N	N Studies
KCNK5	rs10947789	chr6:39174922	1-palmitoylglycerophosphoinositol*	24816252	European	C/T	23	0.0114	0.0043	0.007472	5979	2
			Cholate	24816252	European	C/T	23	0.0296	0.0116	0.01088	5599	2
			Phenyllactate (PLA)	24816252	European	C/T	23	0.0063	0.0027	0.02199	5702	2
			Indoleacetate	24816252	European	C/T	23	0.0078	0.0035	0.02616	7180	2
			Leucine	27005778	European	C/T	28	0.02158	0.01026	0.0356	24724	14
			Pyridoxate	24816252	European	C/T	23	-0.0086	0.0041	0.03579	7263	2
			2-hydroxystearate	24816252	European	C/T	23	0.0053	0.0026	0.04085	7315	2
			1-stearoylglycerophosphoinositol	24816252	European	C/T	23	0.0074	0.0036	0.04098	7251	2
			Isovalerate	24816252	European	C/T	24	0.007	0.0035	0.04308	6735	2
			Isovalerylcarnitine	24816252	European	C/T	23	-0.0069	0.0035	0.04463	7344	2
			Pyruvate	24816252	European	C/T	23	-0.0086	0.0043	0.04471	7237	2
			Hexadecanedioate	24816252	European	C/T	23	-0.0104	0.0052	0.04497	6447	2
CUBN	**rs141640975**	**chr10:16992011**	Creatinine	27005778	European	A/G	0.31	-0.289	0.1178	0.01432	19780	9

EA: Effect allele, OA: Other allele; EAF: Effect allele frequency in %, P: P value, SE: Standard error

References to ESM Tables

[1] Stevens LA, Coresh J, Greene T, Levey AS (2006) Assessing kidney function--measured and estimated glomerular filtration rate. N Engl J Med 354: 2473-2483

[2] Mahajan A, Wessel J, Willems SM, et al. (2018) Refining the accuracy of validated target identification through coding variant fine-mapping in type 2 diabetes. Nature genetics 50: 559-571

III. Electronic Supplementary Material (ESM) Figures

ESM figure 1. Quantile-quantile (QQ) plot for the discovery stage albuminuria ExWAS

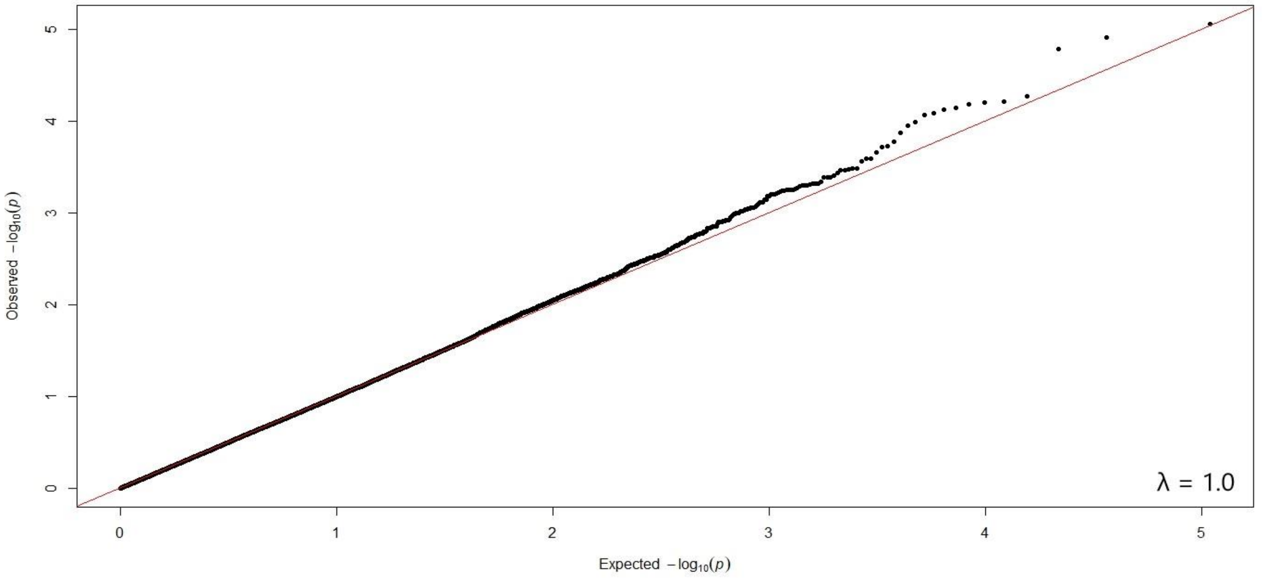

Genomic inflation factor (λ) for the discovery set meta analyses = 1.0

ESM figure 2. Bar plot for *CUBN* rs141640975 genotypes versus mean ACR levels (mg/mmol) in the discovery set.

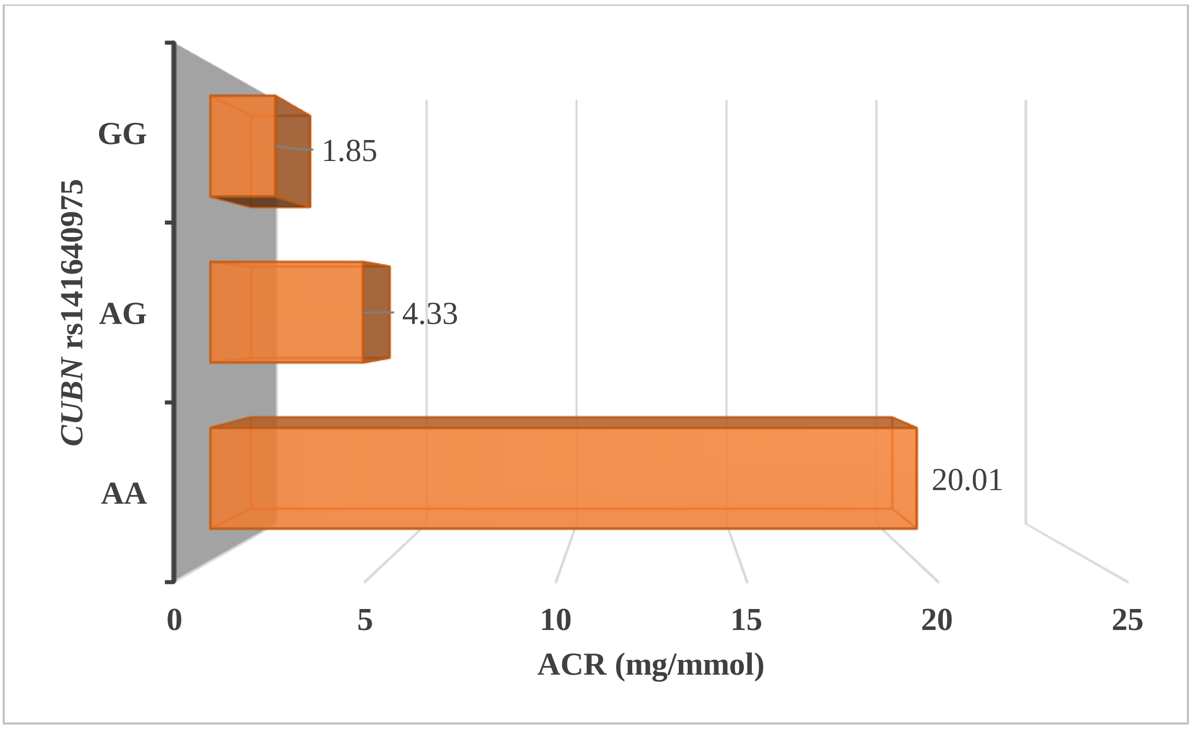

Genotype count for *CUBN* SNP: n_{GG}:13,076, n_{GA}: 218, n_{AA}:2; raw ACR measures (Mean ± SD, mg/mmol), GG: 1.85 ± 10.9; GA: 4.33 ± 37.2; AA: 20.01 ± 18.07

ESM figure 3. Bar plot for *KCNK5* rs10947789 genotypes versus mean ACR levels (mg/mmol) in the discovery set.

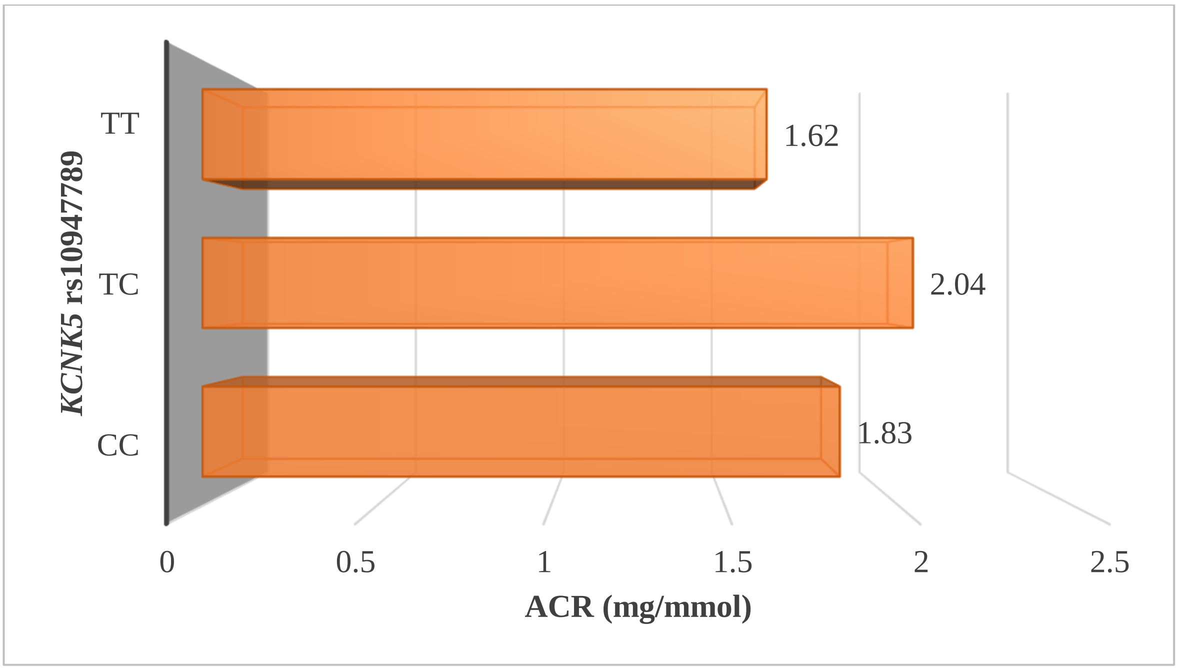

Genotype count for *KCNK5* SNP: n_{TT}: 7,801, n_{TC}: 4,714, n_{CC}: 781; raw ACR measures (Mean ± SD, mg/mmol), TT: 1.62 ± 8.3; TC: 2.04 ± 11.2; CC: 1.83 ± 12.5

ESM figure 4. Bar plot for *LMX1B* rs140177498 genotypes versus mean ACR levels (mg/mmol) in the discovery set.

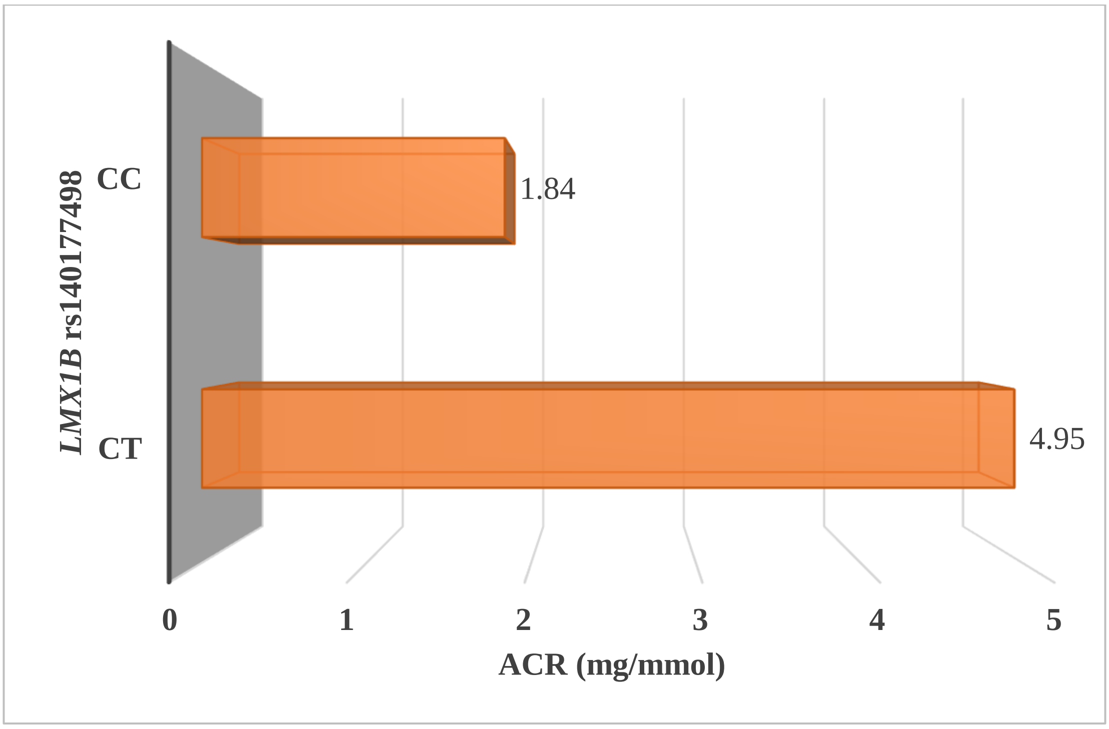

Genotype count for *LMX1B* SNP: n_{CC}: 13,068, n_{CT}: 228, n_{TT}: 0; raw ACR measures (Mean ± SD, mg/mmol), CC: 1.84 ± 11.3; CT: 4.95 ± 28.6;

ESM figure 5. Forest plot of the discovery set meta-analysis for the *CUBN* SNP in pooled group

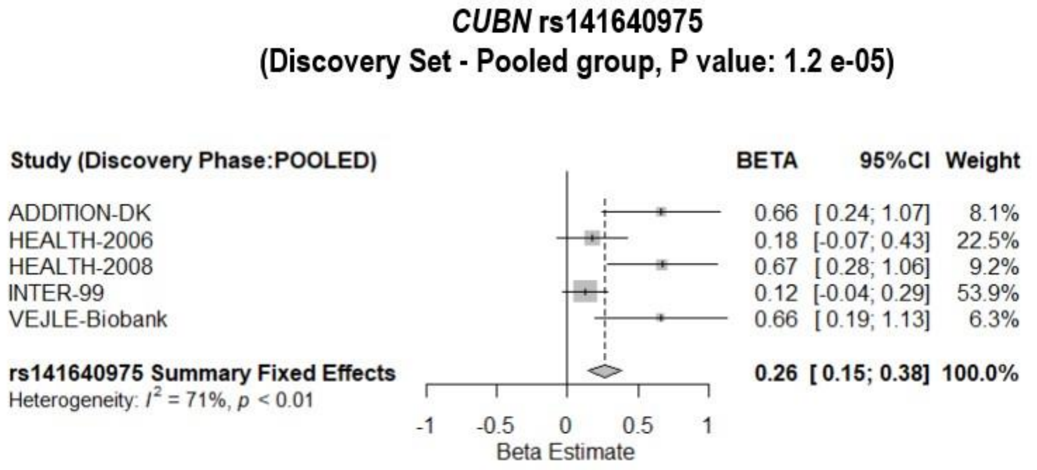

ESM figure 6. Forest plot of the discovery set meta-analysis for the *KCNK5* SNP in pooled group

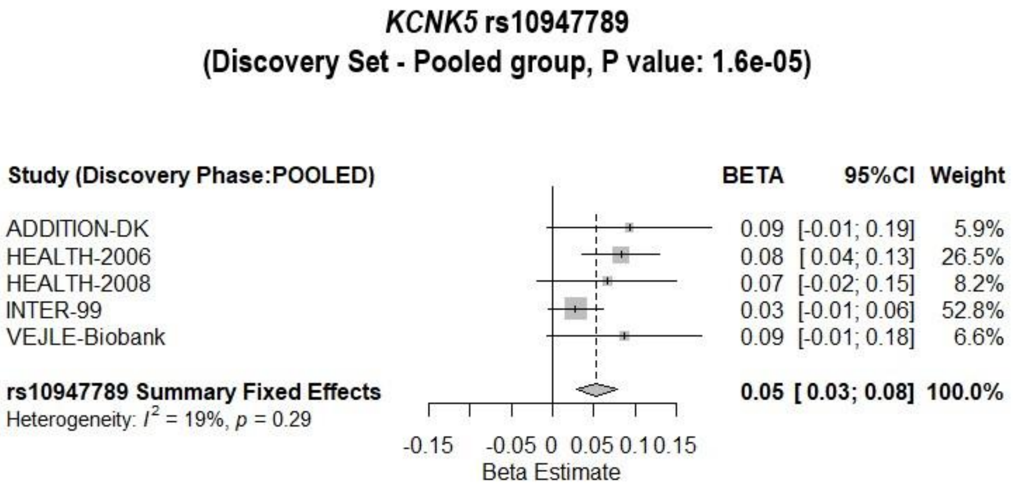

ESM figure 7. Forest plot of the discovery set meta-analysis for the *LMX1B* SNP in pooled group

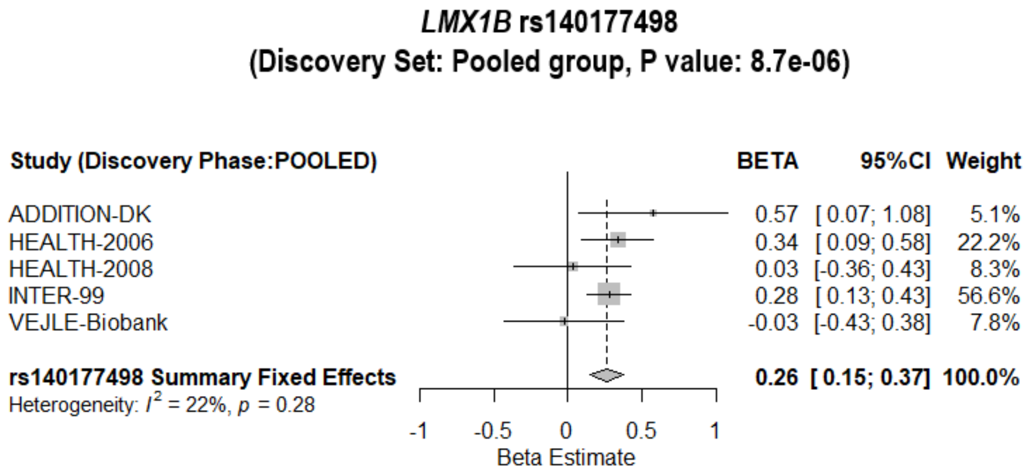

Diabetes Stratified Meta Forest Plots

ESM figure 8. Forest plot of the discovery set meta-analysis for the *CUBN* in diabetes group (DM)

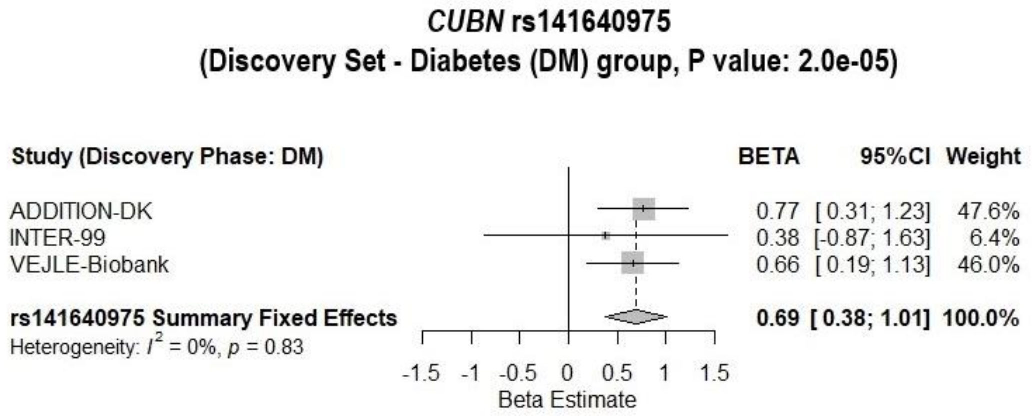

ESM figure 9. Forest plot of the discovery set meta-analysis for the *CUBN* SNP in non-diabetes group (NDM)

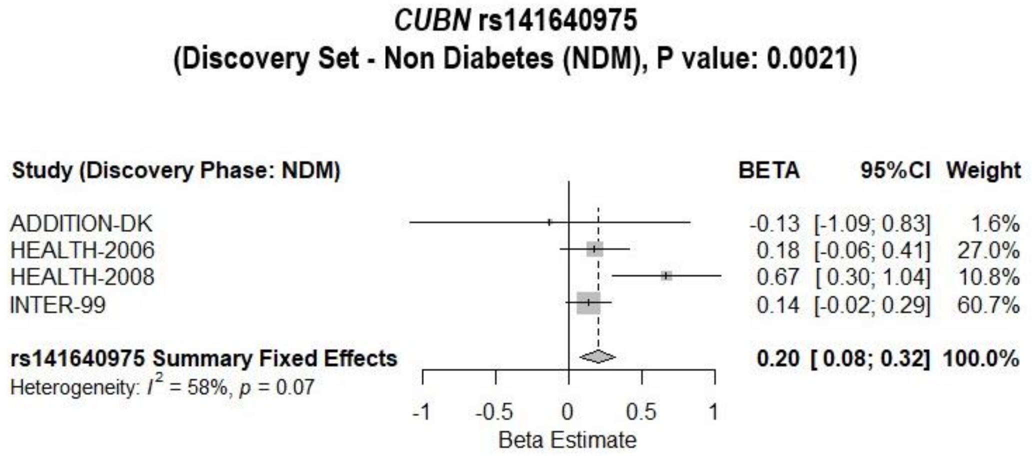

ESM figure 10. Gene aggregate testing: Overall analyses design

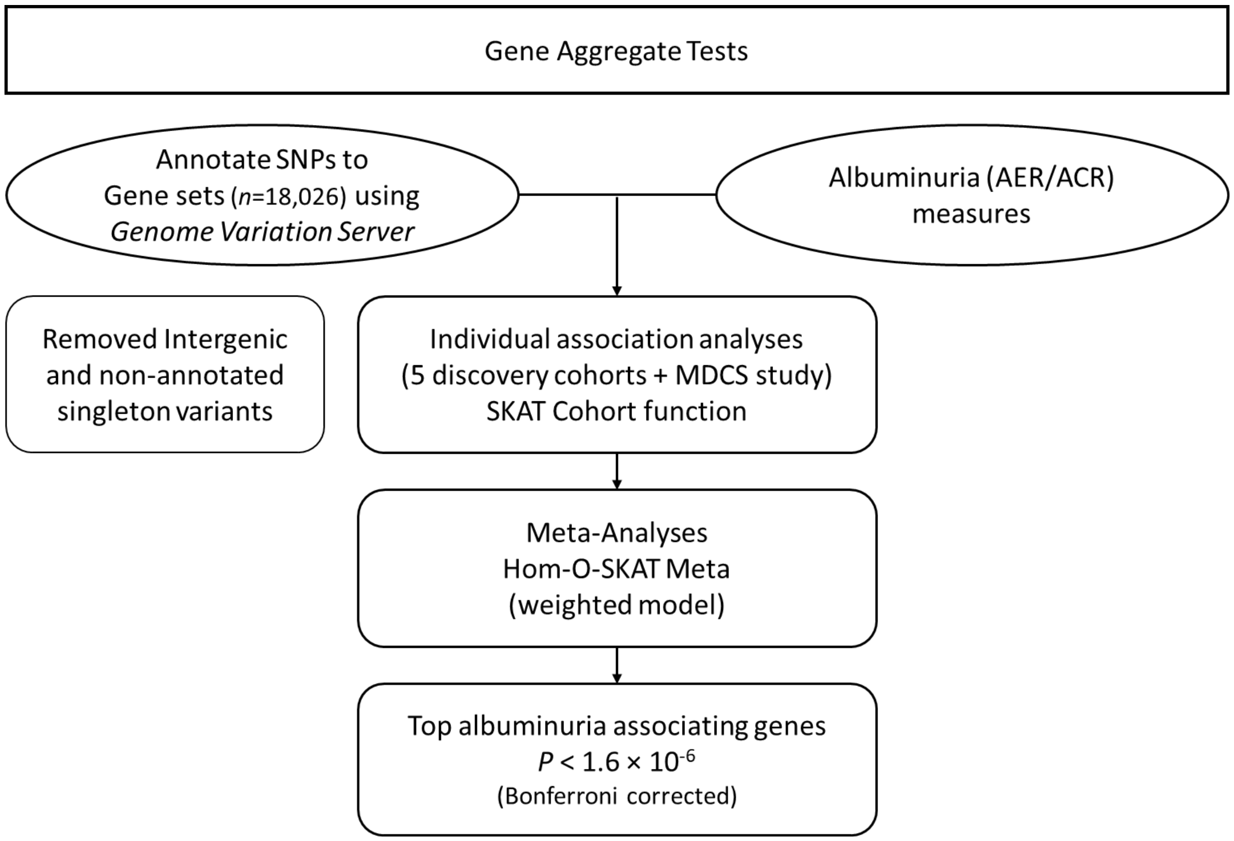

IV. ESM Tables (Funding/Acknowledgements and Study Group members)

Index

ESM Table #	Contents
**11**	Study Acknowledgement and Funding Statement
**12**	IMI-SUMMIT Consortia participants list

ESM table 11 Acknowledgements and Funding Statement

Study Name	Acknowledgements and Funding Statement
**Discovery**	
**Inter99**	This project was funded by the Lundbeck Foundation and produced by The Lundbeck Foundation Centre for Applied Medical Genomics in Personalised Disease Prediction, Prevention and Care (LuCamp, www.lucamp.org). The Novo Nordisk Foundation Center for Basic Metabolic Research is an independent Research Center at the University of Copenhagen partially funded by an unrestricted donation from the Novo Nordisk Foundation (www.metabol.ku.dk). Further funding came from the Danish Council for Independent Research (Medical Sciences).<br>The Inter99 was initiated by Torben Jørgensen (PI), Knut Borch-Johnsen (co-PI), Hans Ibsen, and Troels F. Thomsen. The steering committee comprises the former two and Charlotta Pisinger. The study was financially supported by research grants from the Danish Research Council, the Danish Centre for Health Technology Assessment, Novo Nordisk Inc., Research Foundation of Copenhagen County, Ministry of Internal Affairs and Health, the Danish Heart Foundation, the Danish Pharmaceutical Association, the Augustinus Foundation, the Ib Henriksen Foundation, the Becket Foundation, and the Danish Diabetes Association. We are indebted to the staff and participants of the Inter99, cohort for their important contributions. The authors wish to thank the staff at Novo Nordisk Foundation Center for Basic Metabolic Research, University of Copenhagen, Denmark: A. Forman, T. Lorentzen, B. Andreasen and G. J. Klavsen for technical assistance and A. L. Nielsen, P. Sandbeck and G. Lademann for management assistance.
**Health2006**	The Health2006 study was financially supported by grants from the Velux Foundation; the Danish Medical Research Council, Danish Agency for Science, Technology and Innovation; the Aase and Ejner Danielsens Foundation; ALK-Abello´ A/S (Hørsholm, Denmark), Timber Merchant Vilhelm Bangs Foundation, MEKOS Laboratories (Denmark) and Research Centre for Prevention and Health, the Capital Region of Denmark. This project was also supported by the Lundbeck Foundation and produced by The Lundbeck Foundation Centre for Applied Medical Genomics in Personalised Disease Prediction, Prevention and Care (LuCamp, www.lucamp.org). The Novo Nordisk Foundation Center for Basic Metabolic Research is an independent Research Center at the University of Copenhagen partially funded by an unrestricted donation from the Novo Nordisk Foundation (www.metabol.ku.dk). Further funding came from the Danish Council for Independent Research (Medical Sciences).
**Health2008**	This project was also supported by the Lundbeck Foundation and produced by The Lundbeck Foundation Centre for Applied Medical Genomics in Personalised Disease Prediction, Prevention and Care (LuCamp, www.lucamp.org). The Novo Nordisk Foundation Center for Basic Metabolic Research is an independent Research Center at the University of Copenhagen partially funded by an unrestricted donation from the Novo Nordisk Foundation (www.metabol.ku.dk). Further funding came from the Danish Council for Independent Research (Medical Sciences).
**Vejle biobank**	This project was also supported by the Lundbeck Foundation and produced by The Lundbeck Foundation Centre for Applied Medical Genomics in Personalised Disease Prediction, Prevention and Care (LuCamp, www.lucamp.org). The Novo Nordisk Foundation Center for Basic Metabolic Research is an independent Research Center at the University of Copenhagen partially funded by an unrestricted donation from the Novo Nordisk Foundation (www.metabol.ku.dk). Further funding came from the Danish Council for Independent Research (Medical Sciences).
**ADDITION-DK**	We thank Torsten Lauritzen for access to the data from the ADDITION-Denmark study and also to all the study participants. This project was also supported by the Lundbeck Foundation and produced by The Lundbeck Foundation Centre for Applied Medical Genomics in Personalised Disease Prediction, Prevention and Care (LuCamp, www.lucamp.org). The Novo Nordisk Foundation Center for Basic Metabolic Research is an independent Research Center at the University of Copenhagen partially funded by an unrestricted donation from the Novo Nordisk Foundation (www.metabol.ku.dk). Further funding came from the Danish Council for Independent Research (Medical Sciences). We also thank all the study participants.

Replication	
**MDCS**	This study was supported by the Swedish Research Council, the Swedish Heart and Lung Foundation, the Novo Nordic Foundation, the Swedish Diabetes Foundation, and the Påhlsson Foundation, and by equipment grants from the Knut and Alice Wallenberg Foundation, the Region Skåne, Skåne University Hospital, the Linneus Foundation for the Lund University Diabetes Center and the European Research Council (Consolidator grant nr 649021, Orho-Melander).
**Genesis/GENEDIAB**	Investigators (alphabetical order of the centers; all centers are in France except Liege, which is in Belgium): Diabetology: Amiens (A. Dubreuil, S. Arlot), Angers Coordinating Center (M.Marre, B. Bouhanick, S.Hadjadj, G. Guilloteau), Association Francaise des Diabetiques, Bordeaux-Pessac (H. Gin, V.Rigalleau), Corbeil Essones (G. Charpentier, I. Petit, J.P. Riveline), Grenoble (S. Halimi), Liege (P.J Lefebvre., A.J. Scheen, L.Weekers.), Lille (P. Fontaine, G. Prevot), Lyon-Antiquaille (F. Berthezene, F. Bonnet, S. Marsot), Lyon E-Herriot (C. Thivolet), Marseilles (P. Vague, M.F. Jannot), Nantes (B. Charbonnel, L. Chaillous), Nimes-Montpellier (M.Rodier), Paris-Bichat (M.Marre., E. Larger, A.B.), Paris-Hotel Dieu (G. Slama, J.L. Selam, A. Sola), Paris-Saint Louis (P.Passa, C. Chenu), Poitiers (R.M., F. Torremocha, S.Hadjadj.), Saint Brieuc (C. Colmar-Montiel), Saint Mandé (B.Bauduceau., H. Mayaudon), Strasbourg (M.P. Arpin-Bott), Nancy-Toul (P. Drouin [deceased], P. Mattei, B. Guerci), Toulouse (J.P. Tauber [deceased], H. Hanaire-Broutin, S. Gronier), Valenciennes (O. Verier-Mine).<br>Nephrology: Amiens (A. Fournier, J.M. Achard), Grenoble (D. Cordonnier), Lyon-E Herriot (M. Laville, J.P. Fauvel), Montauban (P. Giraud), Nancy-Vandoeuvre (M. Kessler, E. Renoult), Paris-Necker (J.P. Grunfeld), Poitiers (G. Touchard, F. Bridoux), Reims (J. Chanard, J.P. Melin), Saint Brieuc (C. Charasse).<br>All type 1 diabetes patients and their familiies taking part to the study.<br>This work was supported by grants from the Association Francaise des Diabetiques, INSERM (Institut National de la Sante et de la Recherche Medicale), Societe Francaise d'Hypertension Arterielle, European Union (Euragedic Project; QLG2-CT2001-01 669), Association Diabete et Risque Vasculaire (Paris, France) and JDRF (Juvenile Diabetes Research Foundation).
**DanFunD**	This study was supported by TrygFonden (7-11-0213), the Lundbeck Foundation (R155-2013-14070), and Novo Nordisk Foundation (NNF15OC0015896). The measurements of serum specific IgE were funded by the Lundbeck Foundation (Grant number R165-2013-15410), the A.P. Møller Foundation for the Advancement of Medical Science (Grant number 15-363), and Aase and Einar Danielson's Foundation (Grant number 10-001490). The Novo Nordisk Foundation Center for Basic Metabolic Research is an independent Research Center at the University of Copenhagen partially funded by an unrestricted donation from the Novo Nordisk Foundation (www.metabol.ku.dk).
**Greenlanders**	The Novo Nordisk Foundation Center for Basic Metabolic Research is an independent Research Center at the University of Copenhagen partially funded by an unrestricted donation from the Novo Nordisk Foundation (www.metabol.ku.dk). This project was also funded by the Danish Council for Independent Research (DFF-4090-00244, Sapere Aude grant DFF-11-120909 and DFF-4181-00383), the Steno Diabetes Center Copenhagen (www.steno.dk), the Lundbeck Foundation (R215-2015-4174), and the Novo Nordisk Foundation (NNF15OC0017918, NNF16OC0019986 and NNF15CC0018486). The Greenlandic health surveys (IHIT and B99) were supported by Karen Elise Jensen's Foundation, the Department of Health in Greenland, NunaFonden, Medical Research Council of Denmark, Medical Research Council of Greenland, and the Commission for Scientific Research in Greenland.
**IMI-SUMMIT Consortia**	
Eurodiab, Finn Diane, Cambridge, SDR (Type 1 and 2), Benedict Phase A and B, STENO type 2 diabetes, GoDARTS1,GoDARTS2	This work was supported by the following funders: IMI (SUMMIT 115006); Wellcome Trust 098381, 090532, 106310; NIH R01-MH101814; and JDRF 2-SRA-2014-276-Q-R. SDR: Thanks to Maria Sterner and Malin Neptin for GWAS genotyping in the Scannia Diabetes Registry. Grants: Swedish Research Council, ERC-Adv res grant 269045-GENE TARGET T2D, Academy of Finland grantas 263401 and 267882, Sigrid JUselius Foundation. FinnDiane: We thank M. Parkkonen, A. Sandelin, A-R. Salonen, T. Soppela and J. Tuomikangas for skillful laboratory assistance in the FinnDiane study. We also

	thank all the subjects who participated in the FinnDiane study and gratefully acknowledge all the physicians and nurses at each centre involved in the recruitment of participantsThe FinnDiane Study was supported by grants from the Folkhälsan Research Foundation, the Wilhelm and Else Stockmann Foundation, the Liv och Hälsa Foundation, Helsinki University Central Hospital Research Funds (EVO), the Novo Nordisk Foundation, European Foundation for the Study of Diabetes (EFSD) Young Investigator Research Award funds, and the Academy of Finland (275614 and 299200). GoDARTS: We are grateful to all the participants in this study, the general practitioners, the Scottish School of Primary Care for their help in recruiting the participants, and to the whole team, which includes interviewers, computer and laboratory technicians, clerical workers, research scientists, volunteers, managers, receptionists, and nurses. The study complies with the Declaration of Helsinki. We acknowledge the support of the Health Informatics Centre, University of Dundee for managing and supplying the anonymised data and NHS Tayside, the original data owner. The Wellcome Trust United Kingdom Type 2 Diabetes Case Control Collection (GoDARTS) was funded by The Wellcome Trust (072960/Z/03/Z, 084726/Z/08/Z, 084727/Z/08/Z, 085475/Z/08/Z, 085475/B/08/Z) and as part of the EU IMI-SUMMIT program. The research leading to these results has received funding from the European Union's Seventh Framework Programme (FP7/2007-2013) for the Innovative Medicine Initiative under grant agreement n° 115006

ESM table 12 Members of IMI-SUMMIT Consortium

Serial	Partner	Name	Position
1	1	**Michael Mark**	**Coordinator, WP6 leader**
2	Boehringer-Ingelheim	Markus Albertini	Project manager
3	Ingelheim, Germany	Carine Boustany	Chronic Kidney Disease, Head of Lab
4		Alexander Ehlgen	Transmed
5		Martin Gerl	Biomarker & Bioanlysis, Groupleader
6		Jochen Huber	In vivo Scientist CMDR, Head of Lab
7		Corinna Schölch	Biomarker & Bioanlysis, Head of Lab
8		Heike Zimdahl-Gelling	Pharmacogenomics, Head of Lab
9	2	**Leif Groop**	Prof. Endocrinology; Coordinator Managing entity IMI-JU; PI; **WP1 and WP6 leader**
10	Lund University	Elisabet Agardh	Prof. Ophthalmology
11	Clinical Research Centre	Emma Ahlqvist	Postdoc
12	Malmö, Sweden	Tord Ajanki	Communication strategist
13		Nibal Al Maghrabi	Research nurse
14		Peter Almgren	Biostatistician
15		Jan Apelqvist	Diabetologist
16		Eva Bengtsson	Assis. Prof. Cardiovascular research
17		Lisa Berglund	Postdoc
18		Harry Björckbacka	Assis. Prof. Cardiovascular research
19		Ulrika Blom-Nilsson	LUDC administrator
20		Mattias Borell	Website, server management
21		Agneta Burström	Research nurse
22		Corrado Cilio	Assoc. Prof. Cellular autoimmunity
23		Magnus Cinthio	Assist. Prof. Electrical Measurements, Lund Technical University
24		Karl Dreja	Nephrologist
25		Pontus Dunér	Postdoc Exp. Cardiovasc. Research
26		Daniel Engelbertsen	PhD student Exp. Cardiovasc. Research
27		Joao Fadista	Postdoc
28		Maria Gomez	Assoc. Prof. Cardiovascular disease, **WP4 co-leader**
29		Isabel Goncalves	Assis. Prof. Cardiovascular research
30		Bo Hedblad	Prof. Cardiovascular epidemiology
31		Anna Hultgårdh	Prof. Vessel Wall Biology
32		Martin E. Johansson	Pathologist
33		Cecilia Kennbäck	Laboratory Engineer
34		Jasmina Kravic	Database manager
35		Claes Ladenvall	Genetic statistician
36		Åke Lernmark	Prof. Type 1 diabetes and celiac disease
37		Eero Lindholm	Physician, Researcher Diabetic Complications
38		Charlotte Ling	Assist. Prof. Epigenetics
39		Holger Luthman	Prof. Medical genetics
40		Olle Melander	Assoc. Prof. Hypertension and cardiovascular disease
41		Malin Neptin	Biomedical analyst
42		Jan Nilsson	Prof. Experimental Cardiovascular research, **WP3 leader**
43		Peter Nilsson	Prof. Internal medicine
44		Tobias Nilsson	PhD student Electrical Measurements, Lund Technical University

45		Gunilla Nordin Fredriksson	Prof. Cardiovascular research
46		Marju Orho-Melander	Prof. Genetic epidemiology
47		Emilia Ottoson-Laakso	PhD student
48		Annie Persson	Research nurse
49		Margaretha Persson	Laboratory Engineer
50		Mats-Åke Persson	Database manager
51		Jacqueline Postma	Project manager
52		Elisabeth Pranter	Research nurse
53		Sara Rattik	PhD student Exp. Cardiovasc. Research
54		Gunnar Sterner	Chief physician Internal Medicine Research Unit
55		Lilian Tindberg	Research nurse
56		Maria Wigren	Postdoc Exp. Cardiovasc. Research
57		Anna Zetterqvist	PhD student
58		Mikael Åkerlund	Postdoc
59		Gerd Östling	Laboratory Engineer
60	3	**Timo Kanninen**	Technical director; PI
61	Biocomputing Platforms	Anni Ahonen-Bishopp	Software development manager
62	(BC Platforms)	Anita Eliasson	Financial and administrative director
63	Espoo, Finland	Timo Herrala	System (server) specialist
64		Päivi Tikka-Kleemola	Service manager
65	4	**Anders Hamsten**	Prof. Cardiovascular disease; Atherosclerosis Research Unit; PI
66	Karolinska Institute	Christer Betsholtz	Prof. Vascular biology
67	Stockholm, Sweden	Ami Björkholm	Administrator
68		Ulf de Faire	Professor emeritus Cardiovascular epidemiology
69		Fariba Foroogh	Research engineer
70		Guillem Genové	Scientist
71		Karl Gertow	Research Assist. Prof. Cardiovascular genetics
72		Bruna Gigante	Assoc. Professor Cardiovascular epidemiology
73		Bing He	Postdoc
74		Karin Leander	Assoc. Professor Cardiovascular epidemiology
75		Olga McLeod	Postdoc
76		Maria Nastase-Mannila	Postdoc
77		Jaako Patrakka	Postdoc
78		Angela Silveira	Assoc. Prof. Cardiovascular genetics
79		Rona Strawbridge	Postdoc
80		Karl Tryggvason	Prof. Medical Chemistry
81		Max Vikström	Statistician
82		John Öhrvik	Professor
83		Anne-May Österholm	Postdoc
84	5	**Barbara Thorand**	Nutritional scientist, epidemiologist
85	Helmholtz Centre	Christian Gieger	Statistician
86	Munich, Germany	Harald Grallert	Biologist
87		Tonia Ludwig	Statistician
88		Barbara Nitz	Scientist
89		Andrea Schneider	Data manager
90		Rui Wang-Sattler	Scientist

91		Astrid Zierer	Statistician
92	6	**Giuseppe Remuzzi**	Institute director; PI
93	Mario Negri Institute for	Ariela Benigni	Head of department Molecular Medicine
94	Pharmacological Research	Roberta Donadelli	Scientist
95		Maria Domenica Lesti	Researcher
96	Bergamo, Italy	Marina Noris	Head Laboratory Immunology and genetics of transplantation and rare diseases
97		Norberto Perico	Senior scientist
98		Annalisa Perna	Biostatistician
99		Rossella Piras	Postdoc
100		Piero Ruggenenti	Head of department Renal medicine, Assist. Prof. Nephrology and dialysis
101		Erica Rurali	Postdoc
102	7	**David Dunger (att: Jane Horsford)**	Prof. Paediatrics; PI
103	University of Cambridge	Ludo Chassin	Senior Data Manager
104	UK	Neil Dalton, London	Clinical biochemistry
105		John Deanfield, London	Paediatric cardiology
106		Jane Horsford	PA to Prof. Dunger
107		Clare Rice	Operations manager/financial contact
108		James Rudd	Cardiovascular imaging
109		Neil Walker	Head Data services
110		Karen Whitehead	Technician
111		Max Wong	Postdoc
112	8	**Helen Colhoun**	Prof. Public health and epidemiology; PI; Vice coordinator Managing entity; **WP2 leader**
113		Fiona Adams	
114	University of Dundee	Tahira Akbar	PA to Helen Colhoun
115	Scotland	Jill Belch	Prof. Vasucular disease
116		Harshal Deshmukh	PhD student
117		Fiona Dove	
118		Angela Ellingford	NHS Tayside Diabetic Retinopathy Screening Programme manager
119		Bassam Farran	Statistician
120		Mike Ferguson	Dean of research Biological chemistry and drug discovery
121		Gary Henderson	
122		Graeme Houston	Consultant radiologist/senior lecturer
123		Faisel Khan	Reader, Vascular & Inflammatory Diseases Research Unit
124		Graham Leese	Consultant diabetologist/reader
125		Yiyuan Liu	PhD student
126		Shona Livingstone	Senior statistician
127		Helen Looker	Epidemiologist
128		Margaret McCann	Project assistant
129		Stuart McGurnaghan	Lead data programmer
130		Andrew Morris	Prof. Diabetic medicine
131		David Newton	
132		Colin Palmer	Prof. Pharmacogenomics
133		Ewan Pearson	Consultant diabetologist/senior lecturer
134		Gillian Reekie	Research Nurse

135		Natalie Smith	Research Nurse
136	9	**Angela Shore**	Prof. Cardiovascular Science, PI
137	Peninsula Medical School	Kuni Aizawa	Postdoc
138	Exeter, UK	Claire Ball	Research nurse
139		Nick Bellenger	Cardiologist
140		Francesco Casanova	Associate Research Fellow Vascular medicine
141		Tim Frayling	Prof. Genetics
142		Phil Gates	Senior lecturer Cardiovascular science
143		Kim Gooding	Postdoc Vascular medicine
144		Andrew Hattersley	Prof. Molecular medicine
145		Roland Ling	Consultant opthalmologist
146		David Mawson	Research technician
147		Robin Shandas	Prof. Bioengineering (Colorado)
148		David Strain	Stroke physician, clinical lecturer
149		Clare Thorn	Postdoc Vascular medicine
150	10	**Ulf Smith**	Prof. ; PI
151	University of Gothenburg	Ann Hammarstedt	Researcher Molecular and clinical medicine
152	Sweden	Hans Häring	Prof. University of Tübingen
153		Oluf Pedersen	Prof. Steno Centre, Copenhagen
154		Georgio Sesti	Prof. Universtiy of Catanzaro
155	11	**Per-Henrik Groop**	Prof. Diabetes genetics; PI
156		Emma Fagerholm	MSc; PhD student, genetics
157	Folkhälsan	Carol Forsblom	Clinical coordinator
158	Helsinki, Finland	Valma Harjutsalo	PhD; FinnDiane Co-PI
159		Maikki Parkkonen	Laboratory manager
160		Niina Sandholm	DSc(PhD); GWAS and bioinformatics, FinnDiane Co-PI
161		Nina Tolonen	MD PhD
		Iiro Toppila	BSc, MSc; bioinformatician
		Erkka Valo	MSc; PhD student, bioinformatician
162	12	**Veikko Salomaa**	Prof. Epidemiology; PI; **deputy leader WP2**
163	The National Institute for Health and Welfare	Aki Havulinna	DSc. (tech), statistician
164	Helsinki, Finland	Kati Kristiansson	PhD
165		Pia Okamo	THL press officer
166		Tomi Peltola	PhD
167		Markus Perola	Professor
168		Arto Pietilä	Statistician
169		Samuli Ripatti	Professor, Statistics
170		Marketta Taimi	Research assistant
171	13	**Seppo Ylä-Herttuala**	Prof.; PI; **WP4 leader**
172	University of Eastern Finland	Mohan Babu	PhD student
173	Kuopio, Finland	Marike Dijkstra	PhD student
174		Erika Gurzeler	PhD student
175		Jenni Huusko	PhD student
176		Ivana Kholová	Postdoc
177		Markku Laakso	Prof.
178		Mari Merentie	PhD student

179		Marja Poikolainen	PA Prof Ylä-Herttuala
180	14	**Mark McCarthy**	Prof. Human type 2 diabetes; Oxford Centre for Diabetes, Endocrinology and Metabolism; Wellcome Trust Centre for Human Genetics; PI; **deputy leader WP1**
181	University of Oxford	Will Rayner	Database manager
182	UK	Neil Robertson	Informatics
183		Natalie van Zuydam	Postdoc
184			
185			
186			
187			
188			
189	15	**Claudio Cobelli**	Prof. ; PI; **WP5 leader**
190	University of Padova	Barbara Di Camillo	Assist. Prof.
191	Italy	Francesca Finotello	PhD student
192	-	Francesco Sambo	Postdoctoral fellow
193	-	Gianna Toffolo	Prof.
194	-	Emanuele Trifoglio	PhD student
	-	-	-
195	16	**Riccardo Bellazzi**	Prof. Bioengineering; PI; **deputy leader WP5**
196		Nicola Barbarini	Postdoctoral fellow
197	University of Pavia	Mauro Bucalo	Software engineer
198	Italy	Christiana Larizza	Assist. Prof.
199		Paolo Magni	Assoc. Prof.
200		Alberto Malovini	Postdoctoral fellow
201		Simone Marini	Postdoctoral fellow
202		Francesca Mulas	Postdoctoral fellow
203		Silvana Quaglini	Prof.
204		Lucia Sacchi	Assist. Prof.
205		Francesca Vitali	
206	17	**Ele Ferrannini**	Prof. Medicine; PI
207		Beatrice Boldrini	Postdoctoral fellow
208	University of Pisa	Michaela Kozakova	Senior investigator Medical Pathophysiology
209	Italy	Andrea Mari	Senior researcher Biomedical engineering (ISIB-CNR, Padova)
210		Carmela Morizzo	Biologist, Sonographer Cardiovascular ultrasound
211		Lucrecia Mota	EGIR administrative office
212		Andrea Natali	Assoc. Prof. Medicine
213		Carlo Palombo	Assoc. Prof. Medicine; **deputy leader WP3**
214		Elena Venturi	Researcher
215		Mark Walker	Prof. Molecular diabetic medicine (Univ Newcastle-upon-Tyne)
216	18	**Carlo Patrono**	Prof.Pharmacology; PI
217	Catholic University of Rome	Francesca Pagliaccia	PhD student
218	Italy	Bianca Rocca	Assist. Prof. Pharmacology
219	19	**Pirjo Nuutila**	Prof. ; PI

220	University of Turku	Johanna Haukkala	PhD student
221	Finland	Juhani Knuuti	Prof. ; Director Turku PET Centre
222		Anne Roivainen	Prof.
223		Antti Saraste	Adj. Prof.
224	20	**Paul McKeague**	Prof. Genetic Epidemiology; PI
225	University of Edinburgh	Norma Brown	Research administrator, Public Health Services
226	Scotland	Marco Colombo	Bioinformaticist
227	21	**Birgit Steckel-Hamann**	Deputy coordinator; PI, Manager IMI, LRL
228	Eli Lilly	Krister Bokvist	Biostatistician
229		Sudha Shankar	Diabetologist
230		Melissa Thomas	Translational Science
231	22	**Li-ming Gan**	Prof.; Translational Science Director Cardiovascular Disease; PI, **WP3 leader**
232	AstraZeneca	Suvi Heinonen	PhD, Internal AZ postdoc, Bioscience
233		Ann-Cathrine Jönsson-Rylander	PhD, Assoc. Prof., Team Leader Bioscience, **WP4 leader**
234		Remi Momo	Postdoctoral fellow
235		Volker Schnecke	Informatician Translational Science, **WP5 leader**
236		Robert Unwin	Translational Science Director Diabetic Nephropathy
237		Anna Walentinsson	Geneticist Translational Science
238		Carl Whatling	Bioscientist
239	23	**Everson Nogoceke**	Pre-clinical and clinical aspects of metabolic and vascular disease; PI; **WP2 leader**
240	Roche	Gonzalo Durán Pacheco	Senior Research Statistician
241		Ivan Formentini	Biomarker & Experimental Medicine Leader
242		Thomas Schindler	Pre-clinical and clinical and clinical biomarkers
243	24	**Piero Tortoli**	Professor of Electronics
244	University of Florence	Luca Bassi	Postdoctoral fellow
245		Enrico Boni	Postdoctoral fellow
246		Alessandro Dallai	Postdoctoral fellow
247		Francesco Guidi	Technician
248		Matteo Lenge	PhD student
249		Riccardo Matera	PhD student
250		Alessandro Ramalli	PhD student
251		Stefano Ricci	Assist. Prof.
252		Jacopo Viti	PhD student
253	25	**Bernd Jablonka**	SAD internal IMI coordinator
254	Sanofi-aventis	Dan Crowther	Biomarker researcher
255		Johan Gassenhuber	Biostatistician
256		Sibylle Hess	Biomarker researcher
257		Thomas Hübschle	Pharmacologist Diabetes
258		Hans-Paul Juretschke	Imaging
259		Hartmut Rütten	Head Translational Medicine

260		Thorsten Sadowski	Pharmacologist Diabetes
261		Paulus Wohlfart	Pharmacologist Diabetes
262	26	**Julia Brosnan**	Biochemist, (pre)clinical research CVD, Pfizer US; **WP2 leader**
263	Pfizer	Valerie Clerin	Cardio-renal biologist, WP2
264		Eric Fauman	Computational biologist
265		Craig Hyde	Statistician
266		Anders Malarstig	Human genetics, Pfizer Europé; **WP1 leader**
267		Nick Pullen	Renal Disease Research Director
268		Mera Tilley	
269		Theresa Tuthill	Imaging specialist
270		Ciara Vangjeli	Cardiovascular genetic epidemiologist, Pfizer Europe
271		Daniel Ziemek	Computational biologist
